# Sleep and circadian disturbances in Alzheimer’s disease: clinical assessment, treatable syndromes, and limits of biomarker inference

**DOI:** 10.3389/fneur.2026.1998439

**Published:** 2026-10-28

**Authors:** Xuying Gan, Xilin Zhou, Shuolan Yan, Simin Yang, Zhihua Li

**Affiliations:** 1 Department of Geriatrics, Chongming Hospital Affiliated to Shanghai University of Medicine & Health Sciences, Shanghai, China; 2 The Second School of Clinical Medicine, Southern Medical University, Guangzhou, China; 3 Neurosurgery Center, National Key Clinical Specialty, Engineering Research Center of Diagnostic and Therapeutic Technology and Devices for Cerebrovascular Diseases in Ministry of Education, Guangdong Provincial Key Laboratory on Brain Function Repair and Regeneration, Zhujiang Hospital Institute for Brain Science and Intelligence, Zhujiang Hospital, Southern Medical University, Guangzhou, China

**Keywords:** actigraphy, Alzheimer’s disease, circadian rhythms, dementia care, insomnia, obstructive sleep apnea, polysomnography, sleep disorders

## Abstract

Sleep and circadian disturbances in Alzheimer’s disease (AD) affect patients and caregivers, but clinical action depends on the syndrome, measurement, and outcome under consideration. This narrative review links sleep assessment to indication-based care while distinguishing symptomatic benefit, physiological target engagement, and disease modification. A purposive literature search was supplemented by targeted checks of PubMed-indexed and original publisher records through 4 October 2026. Selected studies are described by population, AD ascertainment, comparator, and measured endpoint rather than assigned formal evidence-certainty grades. Actigraphy characterizes behavioral rest–activity patterns; melatonin timing and temperature rhythms address endogenous circadian physiology under controlled conditions. Polysomnography and quantitative electroencephalography characterize respiratory events, sleep architecture, and oscillatory activity. These measurements inform different questions and do not independently assign AD etiology or cognitive-functional stage. Clinical priorities include confirmed obstructive sleep apnea, cognition-adapted insomnia care, irregular sleep timing, medication-related effects, parasomnias, limb symptoms, and excessive daytime sleepiness. Interpretation incorporates hypoxemia, treatment exposure, comorbidity, caregiver support, and competing diagnoses. Randomized sleep benefits remain distinct from observational cognitive associations or exploratory imaging and cerebrospinal fluid changes. Human and animal studies of arousal circuits, protein dynamics, inflammation, and fluid transport provide mechanistic context, with conflicting results interpreted according to the actual experimental endpoint. The review provides a clinically organized account of selected evidence, not a comprehensive treatment ranking. Sleep care and cognitive-etiologic assessment should proceed in parallel, with follow-up matched to patient function, safety, and the specific treatment goal.

## 1 Introduction

Sleep and circadian disturbances create two different problems in Alzheimer’s disease (AD): they can compromise the daily lives of patients and caregivers, and they can provide information about vulnerable brain systems. These problems overlap, but they do not require the same evidence or the same tests. An individual with clinically significant insomnia or obstructive sleep apnea (OSA) may need treatment irrespective of AD biomarker status. Conversely, an association between a sleep feature measured by electroencephalography (EEG) and amyloid or tau does not establish that changing that feature will slow cognitive decline. Normal aging, neurodegeneration, comorbidity, and the care environment all contribute to the observed sleep phenotype (1–3).

Previous reviews have established bidirectional relationships between sleep and AD pathology, summarized inflammatory and clearance-related mechanisms, and identified sleep as a potential intervention domain (4–6). The remaining clinical problem is how to use this information without confusing a prognostic association, an explanatory mechanism, and an indication for treatment. A sleep measure may be useful for monitoring nocturnal burden while remaining poor at identifying AD etiology. A treatment may improve sleep without changing the underlying neurodegenerative process. These distinctions are necessary when selecting a clinical assessment or interpreting an intervention trial.

This narrative review links disease context to three decisions: which sleep problem requires investigation, which intervention has evidence relevant to that problem, and which biological interpretation the study design supports. Figure 1 separates independently established disease context from sleep assessment and from the outcome of treatment. The intended audience is clinicians working across neurology, sleep medicine, geriatrics, and memory services. The contribution is a clinically organized synthesis of selected evidence, rather than a new staging system or a formal ranking of treatment efficacy.

**Figure 1:**
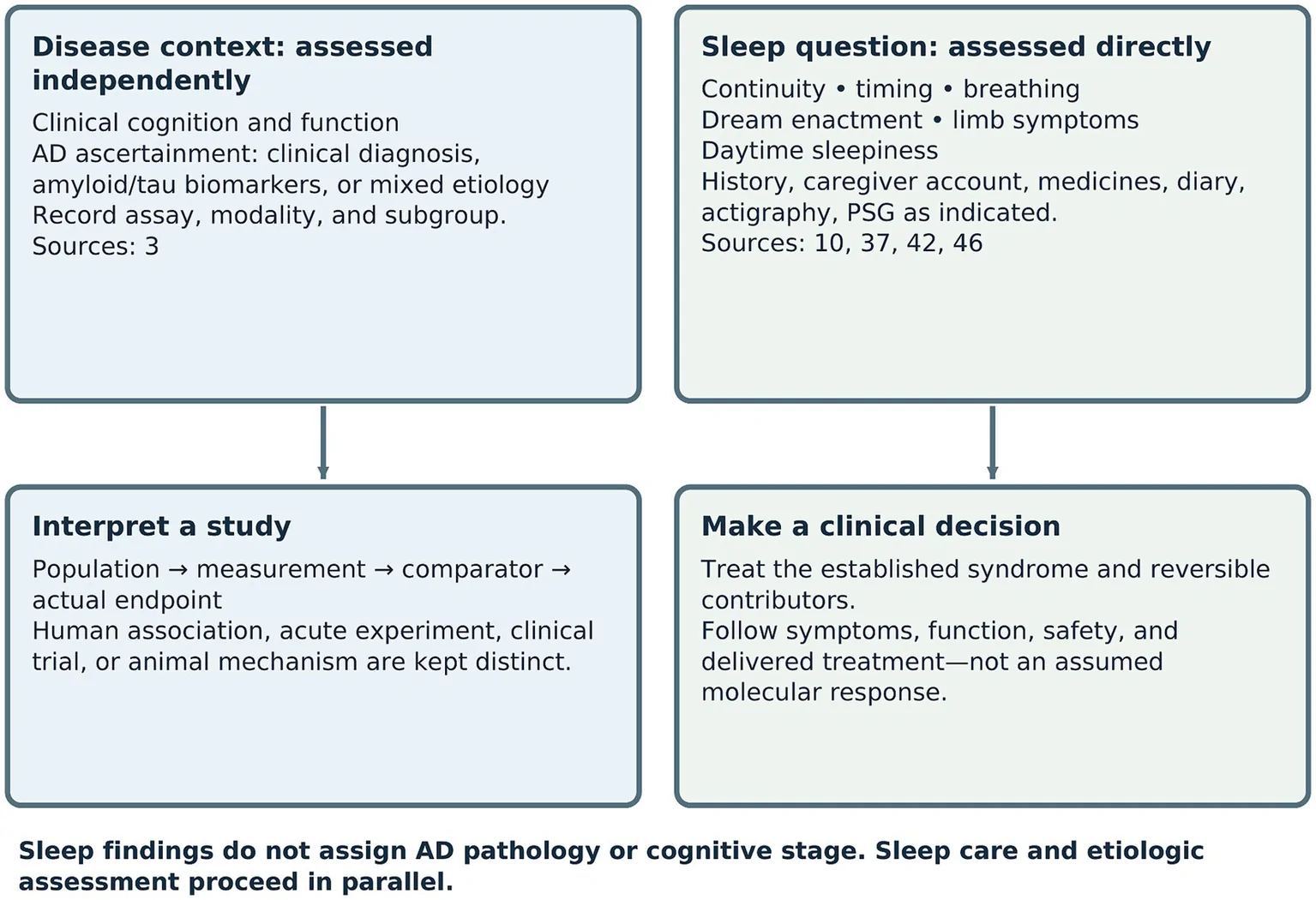
Independently established disease context and symptom-directed sleep care. Clinical cognition/function and AD ascertainment are considered separately from sleep measurements. The two pathways inform study interpretation and clinical action without a sleep-to-stage arrow. This is an organizational diagram, not a validated staging or prescribing algorithm (3, 10, 37, 42, 46). AD, Alzheimer’s Disease; PSG, polysomnography.

Biological AD status and cognitive-functional stage are recorded separately (3). Biomarker characterization is specified as amyloid or tau, cerebrospinal fluid (CSF) or plasma assay, amyloid or tau positron emission tomography (PET), and—where relevant—neuropathology. Amyloid PET, CSF Aβ42 or Aβ42/40, plasma phosphorylated tau, and tau PET are not interchangeable readouts. Whether a biomarker established study eligibility or was only an outcome is also distinguished. A clinically diagnosed probable-AD cohort is therefore not relabeled pathology-confirmed because a subset underwent biomarker testing; mild cognitive impairment (MCI) without etiologic confirmation and mixed/all-cause dementia remain separate contexts. The assay, threshold where available, and clinical group are retained from each source rather than inferred from its title.

## 2 Narrative methods and principles of inference

This purposive narrative review used PubMed/MEDLINE records, original publisher reports, and citation tracing. The retained search history comprises an initial narrative search through 17 July 2026 and focused source-verification updates on 14 September and 4 October 2026. The October update used targeted web queries, direct DOI/PMID retrieval, and cited-reference follow-up to examine clinically relevant 2025–2026 reports on positive airway pressure (PAP), cognition, rest–activity rhythms, AD-related biomarkers, orexin, and sleep-related fluid physiology. Supplementary Table S1 records the available search strings, sources, dates, restrictions, and purposes. These focused checks were not an exhaustive database search; unavailable historical queries and screening yields are not reconstructed.

Sources were selected when they answered a defined assessment or treatment question, supported a numerical or displayed statement, distinguished an AD population from a comparator population, or materially challenged an interpretation. Primary reports were preferred for outcomes and comparators; applicable guidelines informed diagnostic and treatment indications. Reviews supplied broader context, not substitutes for key primary findings. Reports in other disorders were retained when they informed differential diagnosis or exposed limits of generalization. English-language search terms were used, without a publication-date or study-design filter for source verification. Selection did not depend on a favorable result: null randomized endpoints, discordant physiological findings, and adverse effects were retained alongside positive observations.

The intervention table is a set of selected clinical contrasts, not an exhaustive inventory of eligible trials. It includes a direct AD/OSA randomized comparison, an MCI adherence-based comparison, a non-AD randomized counterexample, an AD insomnia trial, dementia-adapted behavioral care, circadian guidance, syndrome-selected restless legs syndrome (RLS) treatment, and experimental physiological studies. These examples distinguish treatment indication from cognitive efficacy and biomarker response. Supplementary Table S2 identifies their source populations, measurements, comparators, outcomes, and reasons for inclusion, together with the principal biomarker studies discussed in the review.

Study design, comparator, endpoint, duration, population relevance, and major limitations are described explicitly. We do not assign evidence-maturity scores, certainty tiers, or comparative treatment rankings. Recommendations attributed to guidelines remain guideline recommendations; the review’s clinical synthesis is identified separately. A null prespecified randomized comparison is not replaced by a positive within-group or responder analysis. There was no prospectively registered review protocol, systematic duplicate screening, new meta-analysis, or formal study-level risk-of-bias instrument. Screening yields and the number of omitted studies cannot be reconstructed from this focused search. The resulting synthesis is consequently informative for clinical reasoning but does not establish the completeness or overall certainty of an intervention evidence base.

## 3 What sleep phenotypes can tell us before and during cognitive decline

### 3.1 Rest–activity measures: longitudinal signals without etiologic specificity

Prospective studies connect disturbed sleep or rest–activity organization with subsequent cognitive decline, but their diagnostic endpoints differ. Actigraphic sleep fragmentation was associated with clinically diagnosed incident AD in older adults, whereas several community cohorts used incident MCI or all-cause dementia (7–9). These findings justify attention to longitudinal change, not an AD diagnosis from a wrist recording. Motor impairment, depression, frailty, social routine, pain, and pre-existing neurodegeneration may influence both movement-derived measures and cognition. Within-person deterioration may be clinically informative even when a population-derived risk estimate cannot be transported to a different patient.

The metrics themselves must be specified. Interdaily stability (IS) describes day-to-day regularity, whereas intradaily variability (IV) reflects within-day rest–activity fragmentation. Relative amplitude contrasts mean activity in the most-active 10-h interval (M10) and least-active 5-h interval (L5). In a cosinor model, mesor is the rhythm-adjusted mean, amplitude is half the modeled peak-to-trough difference, and acrophase is modeled peak timing. These are behavioral rest–activity measures: mobility, frailty, disability, light exposure, caregiver routines, and opportunity for activity can alter them without a corresponding change in the endogenous clock. Sleep–wake timing describes when sleep occurs; entrainment refers to synchronization of endogenous rhythms to environmental time cues. A regular care schedule alone does not demonstrate physiological entrainment. Dim-light melatonin onset and controlled temperature-rhythm assessment address circadian phase more directly, whereas endogenous amplitude requires repeated physiological measurement under appropriate conditions. Serial sampling, lighting control, medications, and care feasibility limit these assessments in dementia. Actigraphy remains practical for multiday patterns but may score immobile wakefulness as sleep (2, 10, 11). Table 1 distinguishes these constructs and measurement limits.

**Table 1:** Specify the sleep or circadian measure before interpreting its clinical meaning.

Measure / construct	What is measured	Clinical or research use	Principal limitation / sources
Sleep duration / time in bed	Report questionnaire, diary, actigraphy, or PSG; distinguish intended sleep opportunity from estimated or recorded sleep.	Clarify insufficient opportunity, prolonged time in bed, and treatment response.	Immobile wakefulness can be scored as sleep; methods are not interchangeable (10, 49)
Continuity / fragmentation	Specify WASO, awakenings, sleep efficiency, EEG arousal index, or movement-derived fragmentation.	Characterize interrupted sleep and investigate its cause.	Respiratory arousals, recalled awakenings, and actigraphic transitions are different endpoints (10, 42)
Respiratory burden	PSG AHI per hour of sleep; HSAT REI per monitoring hour; oxygen depth/duration, arousals, position, and REM context.	Establish OSA and assess control and residual hypoxemia.	AHI alone does not describe total physiological burden or identify AD etiology (41, 42)
N3 / slow-wave activity	N3 is a scored stage; SWA is spectral power in a specified band and montage.	Test an oscillatory target or characterize architecture.	Age, sleep pressure, OSA, medicines, and analytical choices alter both measures (1, 23)
Spindles / coupling	Density, power, amplitude, duration, topography, or slow-oscillation–spindle phase/timing.	Describe sleep-network coordination.	No universal threshold distinguishes aging from MCI or AD (24, 25)
REM architecture / atonia	Separate REM latency and proportion from REM sleep without atonia and dream enactment.	Choose between an architecture question and a parasomnia work-up.	Plasma p-tau181, amyloid PET, and RBD establish different contexts (28, 29, 46)
Behavioral rest–activity rhythms	IS: day-to-day regularity; IV: within-day fragmentation; RA = (M10 − L5)/(M10 + L5); acrophase and mesor are model-specific.	Describe activity organization, timing, and longitudinal change.	Mobility, frailty, lighting, care schedules, device site, and algorithm affect the record (10, 12–14)
Endogenous circadian phase / amplitude	Melatonin timing under dim light or repeated temperature rhythms under controlled conditions; specify protocol and masking.	Investigate suspected phase misalignment or physiological rhythm characteristics.	Acrophase of activity is not DLMO. Serial sampling, environmental control, and medications limit routine dementia use (2, 11)
Entrainment / sleep–wake timing	Record light/activity schedules, sleep opportunity, caregiver routines, and alignment of timing with environment.	Identify modifiable scheduling and light-exposure factors.	Improved behavioral regularity does not alone prove a change in endogenous amplitude or AD pathology (2, 10, 11)

AD, Alzheimer’s disease; AHI, apnea–hypopnea index; DLMO, dim-light melatonin onset; EEG, electroencephalography; HSAT, home sleep apnea testing; IS, interdaily stability; IV, intradaily variability; L5/M10, least−/most-active contiguous 5−/10-h interval; MCI, mild cognitive impairment; N3, non-REM stage 3; OSA, obstructive sleep apnea; PSG, polysomnography; RA, relative amplitude; RBD, REM sleep behavior disorder; REI, respiratory event index; REM, rapid eye movement; SWA, slow-wave activity; WASO, wake after sleep onset.

Figure 2 shows source-specific associations rather than a pooled effect. Winer and colleagues followed 82,829 UK Biobank participants for a mean 6.8 years, with 187 AD outcomes identified through hospital and primary-care records (12). Hazard ratios (HRs) per standard-deviation increase were 1.25 (95% confidence interval [CI] 1.05–1.48) for IS, 0.79 (0.65–0.96) for amplitude, 0.77 (0.59–0.998) for mesor in the published abstract, and 0.75 (0.61–0.94) for M10. The article’s main table reports mesor as 0.78; the abstract–table discrepancy is disclosed in Figure 2 rather than treated as a recalculated estimate. Posner and colleagues studied 1,232 older women using categorical logistic models: earlier versus average L5 midpoint had an odds ratio (OR) of 1.66 (1.08–2.55), and low versus high relative amplitude had OR 1.96 (1.14–3.35), for incident dementia rather than MCI (13). These ORs are not interchangeable with continuous Cox HRs. Posner and Tranah also drew on the same Study of Osteoporotic Fractures cohort, so their reports are not independent cohort replications (9, 13).

**Figure 2:**
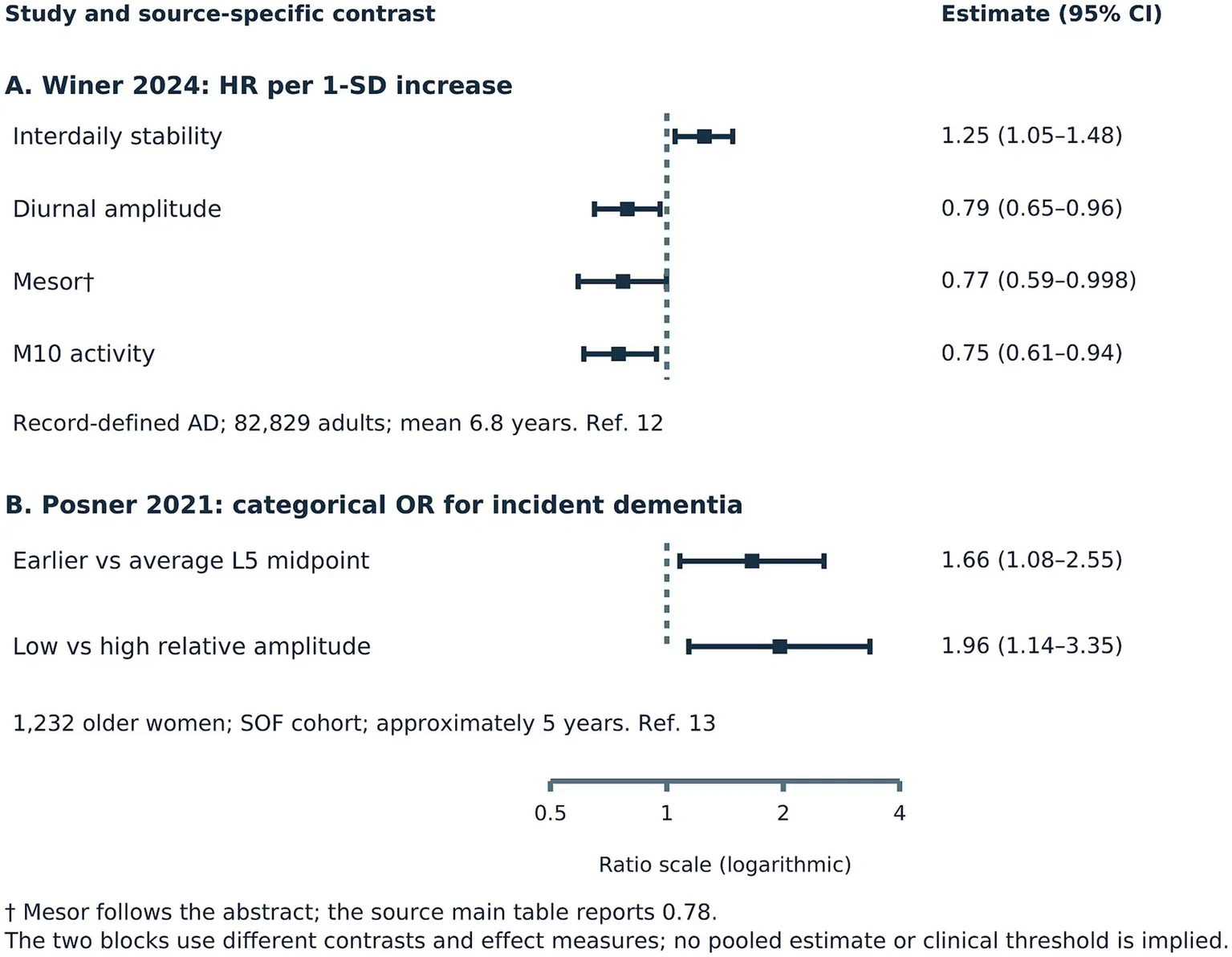
Selected source-specific rest–activity associations. **(A)** Shows *HRs* per one-*SD* increase from Winer et al. in 82,829 UK Biobank participants with 187 record-defined AD outcomes over mean 6.8 years (12). Mesor follows the abstract, *HR* 0.77 (95% *CI* 0.59–0.998); the article’s main table reports 0.78. **(B)** Shows categorical *ORs* for incident dementia in 1,232 older women from Posner et al. (13); these associations should not be relabeled *MCI* outcomes. The two blocks have different contrasts and models. No pooled effect, prediction performance, or clinical threshold is calculated. CI, confidence interval; HR, hazard ratio; IS, interdaily stability; L5, least-active five-hour interval; M10, most-active 10-hour interval; MCI, mild cognitive impairment; OR, odds ratio; SD, standard deviation; SOF, Study of Osteoporotic Fractures.

The unexpected association between higher IS and AD in Winer’s study should not be read as evidence that regular routines are harmful. Reduced activity diversity, rigid daily routines during prodromal disease, residual confounding, and record-based ascertainment are possible explanations rather than demonstrated mediators. Metric and setting matter: the ARIC report in 2,183 older adults linked lower relative amplitude and higher IV to incident dementia (14). A separate ARIC analysis used median absolute deviations to distinguish day-to-day variability in activity volume from variability in timing; their directions differed, and these measures are not conventional IV (15). Chung and colleagues examined adults with Down syndrome cross-sectionally: higher IS related to better cognitive performance, but the reported IV association with MCI/dementia did not survive false-discovery-rate correction (16). That population and design do not directly replicate sporadic incident AD. Each result should therefore retain its metric, unit, diagnostic endpoint, and adjustment context.

Genetic susceptibility supplies another context rather than pathological confirmation. Yang and colleagues examined AD polygenic risk, *APOE* ε4 dose, and accelerometer-derived rhythms in 80,378 UK Biobank participants, with 949 incident dementia and 371 AD records (17). Associations depended on outcome and activity adjustment, and the tested genetic interactions were not significant. Shared UK Biobank participants preclude describing this as external replication of Winer’s cohort; genetic enrichment alone does not establish amyloid or tau status.

Longitudinal sleep trajectories further argue against treating a single baseline measure as a stable exposure. In 1,449 community participants, Zaheed et al. (18) found that sleep amount and regularity changed differently before and after cognitive diagnoses. A duration associated with risk before impairment may have a different interpretation after dementia, when longer sleep estimates or reduced regularity can reflect increasing disease burden.

Biomarker-characterized work supplies a different context. Musiek and colleagues studied 189 cognitively normal adults, relating actigraphic fragmentation to [11C]Pittsburgh compound B amyloid PET and CSF phosphorylated-tau/Aβ42 information in the available subsets (19). Ju et al. (20) related two-week actigraphy and sleep diaries to CSF Aβ42; their assay-specific amyloid classification used Aβ42 ≤ 500 pg./mL. These are pathological contexts for an association, not universal sleep-test thresholds or confirmation that every participant had both amyloid and tau positivity. Supplementary Table S2 records the assay, population, and distinction between biomarker-based eligibility and a biomarker measured as an outcome.

### 3.2 Sleep architecture and microstructure: explain the measure before the mechanism

Polysomnography (PSG) separates sleep stages and detects respiratory events, arousals, and limb movements. Quantitative EEG then characterizes slow-wave activity (SWA), spindle properties, and slow-oscillation–spindle timing. Non-rapid eye movement (NREM) stage N3 is reported as scored minutes or a percentage of total sleep time; SWA is spectral power within a specified low-frequency band. Spindle density, amplitude, duration, sigma power, and coupling precision describe different processes and should not be summarized simply as “spindle expression.” Likewise, wake after sleep onset (WASO), awakening counts, EEG arousal index, and actigraphic fragmentation cannot be treated as equivalent measures of disrupted continuity (1, 21).

Human studies relate oscillatory features to memory and AD-related pathology, but the measurement and sample must remain explicit. Mander and colleagues related amyloid burden to NREM slow waves and hippocampus-dependent memory (22). Lucey et al. (23) recorded home single-channel EEG in 119 older adults, predominantly cognitively normal, with [18F]AV-45 amyloid PET, [18F]AV-1451 tau PET, or CSF Aβ42, total tau, and phosphorylated tau available in different subsets; reduced NREM SWA, particularly at 1–2 Hz, was associated with tau-related measures. The corrected report distinguishes 38 participants in the PET analyses from 23 in the CSF-specific models. Helfrich et al. (24) linked disrupted slow-wave–spindle synchrony to aging, brain atrophy, and forgetting. These findings support sleep-network phenotyping, with age, OSA, medications, and other neurodegenerative processes considered alongside AD pathology.

Negative comparisons are especially informative. Ladenbauer et al. (25) found differences between young adults and older groups during a daytime nap, but no significant difference between healthy older adults and patients with MCI for several measures, including slow-oscillation–spindle coupling. These null group comparisons do not prove equivalence, but they caution against attributing age-related coupling changes specifically to MCI. Nap and overnight recordings, electrode placement, detection algorithms, and the number of usable sleep events further constrain comparison. This evidence favors standardized reporting and age-appropriate comparators rather than a single universal “AD sleep signature.”

Rapid eye movement (REM) findings require equal precision. PSG studies describe REM proportion, latency, and EEG features in MCI and AD; a study in amnestic MCI related REM sleep to magnetic resonance imaging (MRI)-derived cholinergic basal-forebrain volume rather than confirming AD pathology with that volume (26–28). These measures differ from REM sleep without atonia and dream enactment, which raise the diagnostic question of REM sleep behavior disorder (RBD). Jin et al. (29) examined 128 participants across clinical AD, MCI, and normal cognition using PSG, [18F]florbetapir amyloid PET, and plasma p-tau181, neurofilament light, and brain-derived neurotrophic factor. Longer REM latency was associated with selected amyloid and plasma p-tau181 outcomes, whereas slow-wave sleep (SWS) associations were not robust in the adjusted/multiplicity context. This is neither tau-PET evidence nor proof that REM is universally more informative than NREM.

### 3.3 Amyloid and tau associations: timing and compartments matter

Self-reported sleep associations are measure specific. In 70 community-dwelling older adults, Spira et al. (30) associated shorter or poorer reported sleep with greater [11C]Pittsburgh compound B amyloid-PET burden. In 98 cognitively healthy late-middle-aged adults, Sprecher et al. (31) found associations for selected sleep-quality and somnolence measures, but not reported sleep amount or the Epworth Sleepiness Scale. Thus, a positive result for perceived sleep quality cannot be generalized to every duration or sleepiness measure. Acute sleep deprivation also changes CSF amyloid measures or amyloid-PET signal in healthy participants, while sleep–wake manipulation influences human CSF and mouse interstitial-fluid tau (32–34). These acute experiments address state-dependent physiology, not the long-term effect of treating insomnia in biomarker-confirmed AD, and do not independently distinguish production, release, transport, and elimination.

Accordingly, a biomarker study should be interpreted with its sampling time, assay, compartment, and normalization procedure. Repeated associations during longitudinal follow-up are more relevant to accumulation than a single morning concentration, but remain vulnerable to reverse causation. Clinical interpretation should start with the independently established cognitive and etiologic context, then ask what additional information the sleep measure provides. This shifts the question from whether sleep is generally “linked to AD” to whether a specified measurement changes an assessment or meaningfully tests a biological hypothesis.

## 4 Sleep syndromes that generate immediate clinical burden

### 4.1 Insomnia, irregular sleep timing, and the patient–caregiver unit

Sleep complaints are common, but prevalence depends on syndrome definition, informant, care setting, and etiology. In Rongve et al. (35) 151-person early-dementia sample, 71% had at least one caregiver-reported sleep disturbance; insomnia was reported in 29.9% and excessive daytime sleepiness in 22.6%. These are mixed-etiology, informant-based estimates, not PSG-confirmed AD-specific prevalences. In a selected clinic sample of 128 patients with AD, Gaeta and colleagues found an apnea–hypopnea index (AHI) above 5/h in 90.6%, despite a median Epworth Sleepiness Scale score of 5 (36). The threshold and referral setting explain why this cannot be generalized as a population prevalence or used to exclude OSA when sleepiness is absent. Nighttime disturbance affects patient comfort, safety, caregiver sleep, and the sustainability of care. Figure 3 separates patient and caregiver outcomes from reversible contributors without assigning causal effects on institutionalization or mortality.

**Figure 3:**
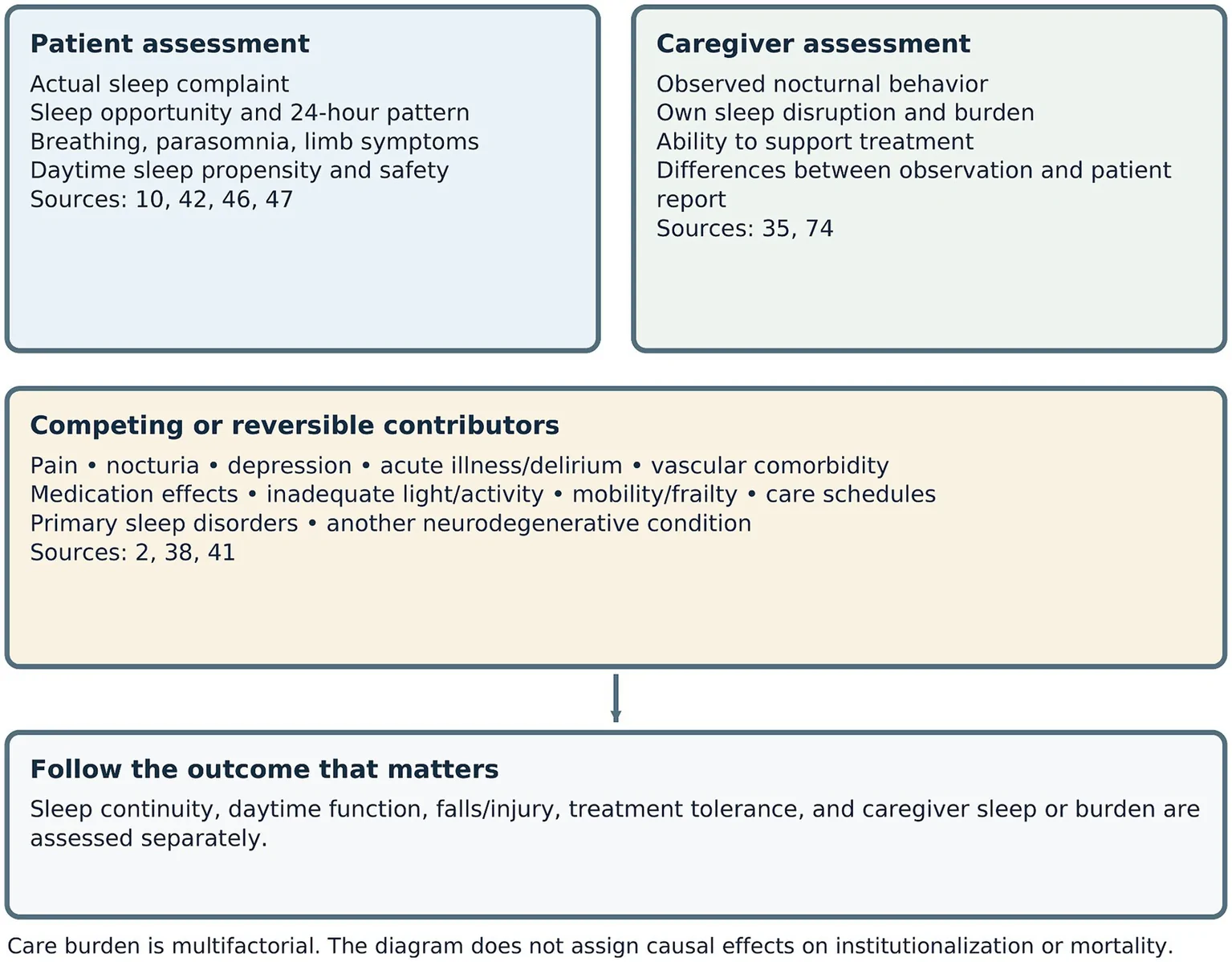
Patient and caregiver burden assessed alongside reversible contributors. Patient symptoms, caregiver observations, implementation capacity, and acute or chronic competing causes are displayed as distinct assessment domains. The figure does not assign independent causal effects on institutionalization, mortality, or AD progression (2, 10, 35, 38, 41, 42, 46, 47, 74). OSA, obstructive sleep apnea; PSG, polysomnography.

Insomnia assessment links difficulty initiating or maintaining sleep to daytime impairment despite adequate opportunity and circumstances for sleep; nighttime wakefulness alone is insufficient for diagnosis. In advanced cognitive impairment, patient self-report may be unreliable, and collateral observations must be interpreted alongside daytime function, sleep opportunity, and competing causes. An abrupt change in nighttime behavior warrants assessment for an acute medical or medication-related cause before it is labeled progression of a chronic sleep disorder. Persistent symptoms require review of pain, nocturia, breathing disturbance, mood, time spent in bed, activity, and the timing of prescribed and nonprescribed medicines (37, 38).

Irregular sleep–wake rhythm disorder is also distinct from simply sleeping poorly. Actigraphy and a caregiver log can reveal the distribution of sleep and activity across the full day; PSG on one night cannot characterize a multiday timing pattern. Conversely, actigraphy cannot establish sleep stages or exclude OSA. Evening agitation, often called sundowning, is a descriptive behavioral pattern and should not automatically be equated with a circadian phase disorder. Timed-light treatment requires a timing hypothesis, whereas routine daytime activity and support for consistent sleep opportunity address broader environmental contributors (10, 11). Table 2 outlines when to escalate from history and logs to targeted testing.

**Table 2:** Presenting sleep concern, differential diagnosis, and assessment that can change care.

Presenting concern	Clinical differentiation	Useful assessment / escalation	Management and follow-up
Persistent insomnia	Daytime consequences despite adequate opportunity; distinguish pain, nocturia, mood symptoms, delirium, medication timing, and respiratory events.	Patient/caregiver history and diary; actigraphy for unclear timing; PSG when another sleep disorder is suspected.	Cognition-adapted behavioral care; review medicines and monitor sleep plus daytime function (10, 37, 38, 74)
Irregular sleep–wake pattern	Multiday disorganization differs from evening agitation, a phase disorder, or one poor night.	24-h diary and multiday actigraphy; assess light, activity, and care schedule; specialist phase assessment when indicated.	Daytime activity/light and feasible routines; no universal evening light or melatonin schedule (10, 11)
Possible OSA	Snoring, witnessed pauses, choking, unexplained dozing, vascular risk; absence of reported sleepiness does not exclude OSA.	HSAT in suitable uncomplicated adults; PSG for significant comorbidity, complex disorders, or inadequate/negative HSAT.	Confirm OSA; monitor PAP coverage, residual events, oxygenation, tolerance, and caregiver burden (36, 41–43)
Dream enactment / injury	Differentiate RBD, respiratory-event-related behaviors, other parasomnias, drugs, and possible synucleinopathy.	Bed-partner history, medication review, neurological assessment, and video-PSG.	Safety measures and disease-specific reassessment; do not assume typical AD progression (45, 46)
Evening limb symptoms	Clinical RLS pattern versus cramps, pain, akathisia, neuropathy, or observed movements alone.	History, iron status, medication review; leg EMG/PSG when PLMD or a competing disorder requires assessment.	Treat the defined syndrome and reassess benefit and harms (47, 48)
Excessive daytime sleepiness	Actual propensity to fall asleep versus fatigue, apathy, sedation, inactivity, prolonged time in bed, or insufficient sleep.	Observed episodes, Epworth when reliable, diary; test suspected OSA; specialist review for persistent unexplained hypersomnolence.	Correct the identified cause; monitor alertness and safety, not just recorded sleep duration (42, 49, 50)
Abrupt nocturnal change	Consider delirium, infection, pain, urinary symptoms, new medicines, or acute vascular/systemic illness.	Time course, collateral baseline, physical assessment, and targeted investigation.	Address acute and reversible causes before assigning deterioration to chronic AD or a sleep mechanism (38, 41)

These are clinical assessment priorities, not a validated decision algorithm. AD, Alzheimer’s disease; EMG, electromyography; HSAT, home sleep apnea testing; OSA, obstructive sleep apnea; PAP, positive airway pressure; PLMD, periodic limb movement disorder; PSG, polysomnography; RBD, REM sleep behavior disorder; RLS, restless legs syndrome.

### 4.2 OSA: a treatable comorbidity, not a proven AD-prevention intervention

OSA is important because obstructive events and nocturnal hypoxemia are measurable and treatable. Prospective studies associate sleep-disordered breathing with later cognitive impairment, while Alzheimer’s Disease Neuroimaging Initiative (ADNI) analyses link reported OSA to amyloid-PET and CSF Aβ/tau trajectories (39, 40). Such associations warrant assessment but do not show that PAP prevents AD. AHI counts events, not their cumulative physiological burden. Reports should also describe oxygen-desaturation depth and duration, time below a stated saturation threshold, arousals, sleep fragmentation, REM/supine dependence, and vascular comorbidity where measured. Baseline sleepiness and cognitive stage affect symptom reporting and the room for improvement; a low sleepiness score does not exclude substantial OSA. The American Thoracic Society (ATS) workshop report emphasizes these heterogeneous mechanisms and measurement limits (41).

Questionnaires estimate OSA risk; they do not establish the diagnosis. PSG or technically adequate home sleep apnea testing (HSAT) is appropriate for uncomplicated adults whose symptoms indicate increased risk of moderate-to-severe OSA. PSG is preferred over HSAT in significant cardiorespiratory disease, suspected hypoventilation, neuromuscular respiratory weakness, chronic opioid exposure, a history of stroke, or severe insomnia. A single negative, inconclusive, or technically inadequate HSAT should be followed by PSG (42). Cognitive impairment does not by itself determine the test, but sensor tolerance, comprehension, and caregiver assistance affect feasibility. Reports should distinguish the PSG apnea–hypopnea index, based on sleep time, from a home-test respiratory event index based on monitoring time, and include hypoxemia and the clinical presentation.

Treatment follow-up should document objective nightly PAP use, the proportion of sleep covered, mask leak, residual events or hypoxemia, tolerability, and patient-relevant function (43). Hours of use, a threshold such as 4 hours per night, and coverage of the whole sleep period are different exposure measures; a study adherence threshold is not a proven dose for preventing AD. MCI may permit patient-led use with reminders, whereas established dementia can require caregiver assistance with mask application, cleaning, troubleshooting, and maintenance of routine. Mask discomfort, nocturnal removal, and competing sedative or vascular exposures should be recorded. An adherence-associated cognitive trajectory remains vulnerable to selection and healthy-adherer effects. Treatment of symptomatic OSA does not depend on demonstrating an AD-prevention benefit (41, 44).

### 4.3 Dream enactment and limb symptoms

Prominent dream enactment warrants attention to injury risk and diagnostic reassessment. Video-PSG helps establish REM sleep without atonia and distinguish RBD from respiratory-event-related behaviors or other nocturnal events. In a person labeled as having AD, confirmed RBD should prompt consideration of Lewy body disease or mixed pathology rather than being assumed to represent typical AD progression (45, 46). Immediate priorities include a safe sleep environment and review of relevant medications and comorbidities. The indication for specialist assessment is clinical; it does not depend on demonstrating a specific AD-related circuit lesion.

Restless legs syndrome (RLS) is a clinical syndrome involving an urge to move, usually with discomfort, worsening at rest and in the evening, and relief with movement. Periodic limb movements on a recording are not sufficient to diagnose periodic limb movement disorder; symptoms and exclusion of alternative explanations matter (10, 47). In cognitively impaired patients, descriptions may be limited, making observation, caregiver history, iron assessment, and medication review important. In a randomized trial of 147 older adults with clinically diagnosed AD dementia, nighttime agitation, and RLS, gabapentin enacarbil improved nighttime agitation and actigraphic sleep relative to placebo. Adverse events were common, and a nonsignificant trend toward more falls warrants attention (48). This supports assessing a specific comorbidity rather than treating unexplained agitation empirically as RLS.

### 4.4 Excessive daytime sleepiness: define the complaint before ordering a test

Excessive daytime sleepiness is increased sleep propensity during the intended waking period. It differs from fatigue, low motivation or apathy, inactivity, drug-induced sedation, prolonged time in bed, and insufficient or fragmented nocturnal sleep. Collateral descriptions should identify actual dozing, its timing and consequences, and changes after a new medication or illness. An Epworth score can supplement history when reporting is reliable, but a low score does not rule out OSA in cognitive impairment. A diary or actigraphy can clarify sleep opportunity and timing; respiratory testing addresses suspected OSA. Persistent unexplained hypersomnolence warrants specialist evaluation after insufficient sleep, medicines, and comorbidity have been assessed (36, 41, 42, 49).

Carvalho et al. (50) followed amyloid-PET change in 283 older adults without dementia; baseline sleepiness was associated with subsequent regional amyloid accumulation. This is longitudinal observational evidence, not a trial showing that treating sleepiness lowers amyloid. In MCI, associations of excessive sleep with decline can also reflect illness, depression, OSA, or inactivity (49). Evaluation should therefore seek an actionable cause and measure daytime function rather than prescribe sleep restriction solely because long sleep is associated with risk.

### 4.5 Medication effects and a broader differential diagnosis

Medication review belongs in every sleep assessment, with attention to indication, dose, administration time, recent changes, interactions, and renal or hepatic vulnerability. Donepezil studies show that increased REM proportion can coexist with reduced sleep continuity; a change in architecture is not automatically a sleep benefit (51). Cholinesterase-inhibitor-related insomnia or vivid dreams should prompt review of timing and tolerability with the prescriber, not automatic discontinuation. Memantine can contribute to dizziness, confusion, or somnolence in some patients, but its cognitive indication is separate from sleep treatment (52).

Antidepressants can alter REM expression and, in susceptible patients, contribute to dream enactment or limb symptoms. Benzodiazepines, Z-drugs, sedating antipsychotics, opioids, and anticholinergic drugs require attention to daytime sedation, confusion, falls, and respiratory vulnerability; disease-specific exceptions and goals of care must be considered rather than treating all central nervous system (CNS)-active drugs as interchangeable (38, 46, 47). Table 3 organizes these effects and review priorities. Abrupt sleep or behavioral change also warrants evaluation for delirium, infection, pain, nocturia, depression, vascular illness, environmental disturbance, primary sleep disorders, or another neurodegenerative diagnosis. Treating a reversible contributor is not contingent on proving an AD-related mechanism.

**Table 3:** Medication-related sleep effects and review priorities in cognitive impairment.

Medication group	Potential sleep / behavioral effects	What to review	Interpretive boundary / sources
Cholinesterase inhibitors; donepezil evidence	REM proportion can increase while sleep continuity worsens; insomnia or vivid dreams can be clinically relevant.	Dose and administration time, temporal relation to symptoms, nightmares, tolerability, and underlying cognitive indication.	Architecture change is not a sleep-treatment benefit. Published donepezil findings do not establish identical effects for every agent (51)
Memantine	Somnolence, dizziness, or confusion can complicate sleepiness and safety assessment in some patients.	Dose/titration, renal function, interacting medicines, new daytime symptoms, and treatment indication.	Not an established sleep therapy; do not attribute every sleep complaint to memantine (52)
Antidepressants	Agent-dependent activation or sedation, REM changes, dream enactment, or limb symptoms.	Specific agent, timing, dose changes, depression, parasomnia history, and RLS mimics.	Treat the underlying indication; specialist review may be needed for drug-related RBD (38, 46, 47)
Benzodiazepines / Z-drugs	Sedation, confusion, falls, and impaired daytime performance; additive respiratory risk with other depressants.	Indication, duration, falls/delirium history, OSA or hypoventilation, co-prescribed opioids, and withdrawal risk.	Avoid routine empiric sedation; selected disorder-specific indications require individual assessment (38, 42, 46)
Sedating antipsychotics / anticholinergics	Somnolence, cognitive burden, orthostasis, falls, and altered motor or nocturnal behavior.	Actual psychiatric/safety indication, cumulative anticholinergic load, extrapyramidal symptoms, and non-drug alternatives.	Sedation is not restorative sleep or disease modification; dementia-specific risks must be weighed (38)
Opioids / combinations of CNS depressants	Respiratory depression, sleep-disordered breathing, sedation, and fragmented sleep.	Pain control, dose and combinations, respiratory history, oxygenation, and need for PSG rather than HSAT.	Do not infer adequate ventilation from subjective improvement in sleep (38, 42)
Sleep-directed pharmacotherapy	Effects are drug-, syndrome-, dose-, and population-specific.	Next-day alertness, falls, interactions, response to behavioral care, and the goal of treatment.	Short-term insomnia benefit does not establish class-wide efficacy or slowed AD (38, 75)

The table summarizes clinical review considerations, not instructions to start, stop, or switch a medicine. Changes require the prescribing clinician’s assessment of indications, withdrawal risk, comorbidity, and goals of care. AD, Alzheimer’s disease; CNS, central nervous system; HSAT, home sleep apnea testing; OSA, obstructive sleep apnea; PSG, polysomnography; RBD, REM sleep behavior disorder; REM, rapid eye movement; RLS, restless legs syndrome.

## 5 What mechanisms explain—and what they do not establish

### 5.1 Circuit vulnerability as an explanation for heterogeneity

Sleep regulation depends on distributed timing, arousal, inhibitory, and memory networks. Figure 4 identifies evidence sources for these systems without assigning a sleep feature to a disease stage. Human postmortem work describes suprachiasmatic-nucleus abnormalities, wake-promoting neuronal loss, and associations of sleep fragmentation with ventrolateral preoptic/intermediate-nucleus neuron counts (53–55). Pathological associations provide a substrate for both impaired wake maintenance and disrupted continuity; tissue measurements and sleep recordings are not necessarily contemporaneous.

**Figure 4:**
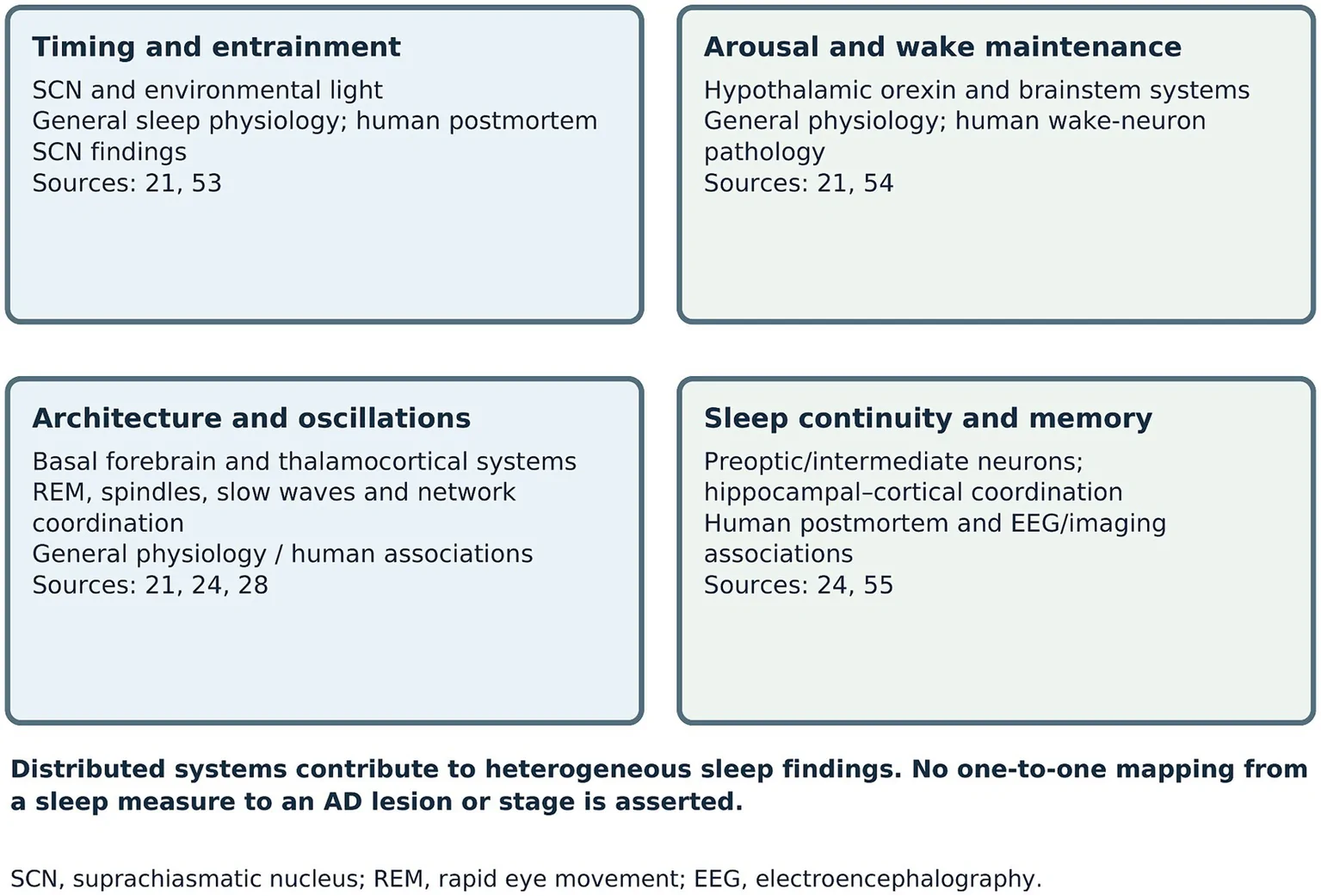
Distributed neural systems and the provenance of supporting evidence. General sleep physiology (21), human SCN and wake-neuron pathological findings (53, 54), preoptic/intermediate-neuron associations (55), and EEG/imaging or REM/basal-forebrain associations (24, 28) are labeled separately. The diagram does not infer uniform TRN suppression or a direct treatment effect on memory. Connections are not an AD-specific causal circuit. EEG, electroencephalography; REM, rapid eye movement; SCN, suprachiasmatic nucleus; TRN, thalamic reticular nucleus.

Orexin illustrates this distinction. It is an arousal regulator, but clinical sleepiness, CSF orexin concentration, and loss of orexin-related neurons are different readouts. Human CSF studies report associations that vary with diagnostic context, while postmortem work demonstrates vulnerability of wake-promoting systems (54–57). Neither a high nor a low orexin value provides a universal insomnia-treatment rule. The clinical evidence for a dual orexin receptor antagonist is strongest when a particular drug is tested for insomnia in a defined population; that indication must be separated from its potential effects on AD pathology.

Basal-forebrain cholinergic networks, thalamocortical circuits, and hippocampal–cortical coordination provide physiological context for REM, spindle, and slow-wave findings (21, 24, 28). Figure 4 therefore depicts distributed functional relationships and distinguishes general sleep physiology from human AD-related observations. An individual spindle abnormality reflects network function and cannot identify uniform thalamic reticular nucleus suppression or a single AD-specific lesion.

### 5.2 Protein dynamics, fluid physiology, inflammation, and vascular injury

Sleep–wake activity and orexin manipulations influence amyloid dynamics in experimental systems, and chronic disruption can worsen pathology in susceptible models (58–60). Figure 5 separates human physiology from animal transport and inflammatory experiments. Reciprocal relationships are plausible, but an acute concentration change, tracer movement, and years-long pathological accumulation are different endpoints. Connections in the figure identify related questions and their sources rather than a single established causal chain.

**Figure 5:**
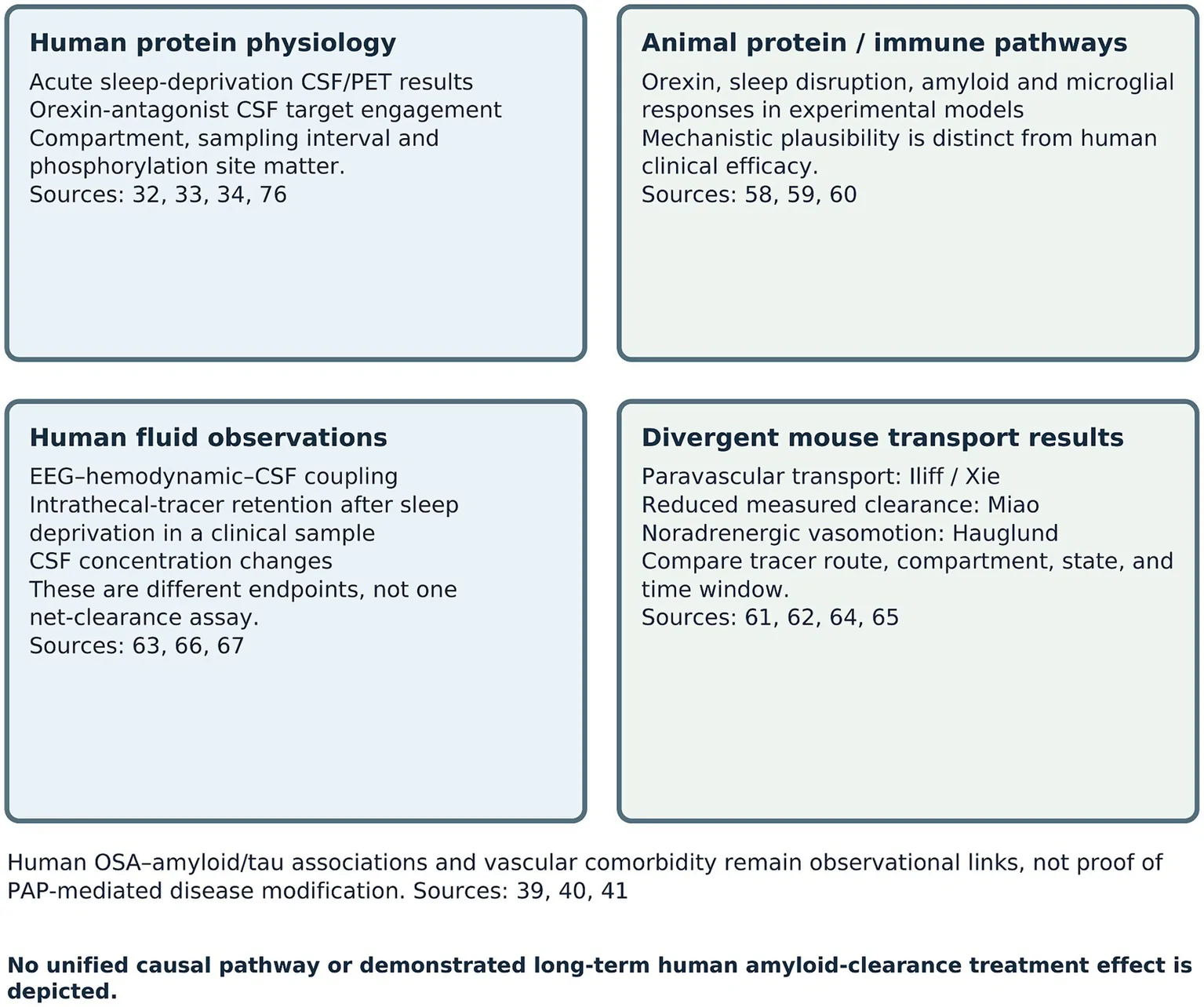
Protein dynamics and fluid physiology separated by species, compartment, and endpoint. Human acute CSF/PET and orexin-antagonist experiments (32–34, 76) are distinguished from animal protein/inflammatory studies (58–60), human coupled CSF physiology and intrathecal-tracer observations (63, 66, 67), and divergent mouse transport experiments (61, 62, 64, 65). Human OSA–cognitive and biomarker associations remain observational (39–41). The layout does not depict a single causal chain or equate CSF oscillation, concentration, retention, and net solute clearance. CSF, cerebrospinal fluid; EEG, electroencephalography; OSA, obstructive sleep apnea; PET, positron emission tomography.

Fluid physiology illustrates why experimental endpoints must remain distinct. Xie and Iliff described sleep-associated or paravascular transport in mice; Fultz showed coupling of EEG, hemodynamic, and CSF oscillations in humans (61–63). Miao’s mouse experiment instead reported reduced measured clearance during sleep and anesthesia (64), whereas Hauglund related NREM noradrenergic vasomotion to transport in mice (65). Human intrathecal-tracer MRI after sleep deprivation found greater tracer retention in a clinical sample (66). Tracer route, compartment, anesthesia, sampling interval, and whether the endpoint measures influx, redistribution, retention, or clearance can account for divergent findings. The human tracer result is more direct than a CSF oscillation for that transport question, but it does not establish normal long-term amyloid removal in all patients with AD. CSF protein concentration changes in healthy-adult sleep experiments likewise are not direct clearance rates (67).

Inflammation and vascular injury provide additional explanations rather than a separate clinically validated sleep-targeting pathway. Sleep loss influences immune signaling, and AD models demonstrate interactions with microglial responses; OSA adds intermittent hypoxemia and vascular stress (39, 60, 68, 69). In clinical cohorts, a common vascular or inflammatory cause could contribute to both sleep disturbance and cognitive impairment. Treating established respiratory or vascular comorbidity is defensible on its own indications. It should not be presented as proof that a sleep-specific inflammatory or blood–brain barrier mechanism has been reversed.

The causal question is consequently design-specific. A cross-sectional association cannot separate prodromal disease causing sleep change from sleep contributing to progression. A long diagnostic lag reduces, but does not eliminate, prodromal bias. Repeated sleep and biomarker measurements can strengthen temporal inference, yet treatment effects still require an adequate comparator. For intervention studies, the interpretive sequence is intervention delivery, physiological target engagement, patient-relevant benefit, and—only when directly evaluated—effects on AD biology and sustained clinical progression. Each step can succeed while the next remains unproven.

## 6 From assessment to treatment: matching the endpoint to the decision

### 6.1 Select the test that can change care

A practical assessment starts by identifying the dominant concern: disturbed continuity, altered timing, breathing events, abnormal behaviors, or daytime sleepiness. Patient and caregiver accounts should be reconciled rather than averaged. A diary establishes timing and opportunity; actigraphy adds repeated activity-derived estimates; PSG answers architecture, respiratory, and movement questions; and video-PSG is particularly relevant to injurious or diagnostically uncertain nocturnal behavior (10, 42, 46). Quantitative EEG or AD biomarkers are not routine substitutes for this clinical sequence.

Figure 6 presents symptom-directed sleep care and cognitive-etiologic evaluation as parallel activities. Neither actigraphy nor a sleep complaint assigns MCI, dementia, or AD pathology. Confirmed OSA, persistent insomnia, and injurious nocturnal behavior can be assessed while biomarker status remains uncertain. Cognitive stage informs communication, consent, caregiver implementation, and safety; it does not mandate a specific drug. Research studies requiring independently established AD biology have separate eligibility and endpoint requirements. The diagram provides an assessment structure, not a validated diagnostic or prescribing algorithm.

**Figure 6:**
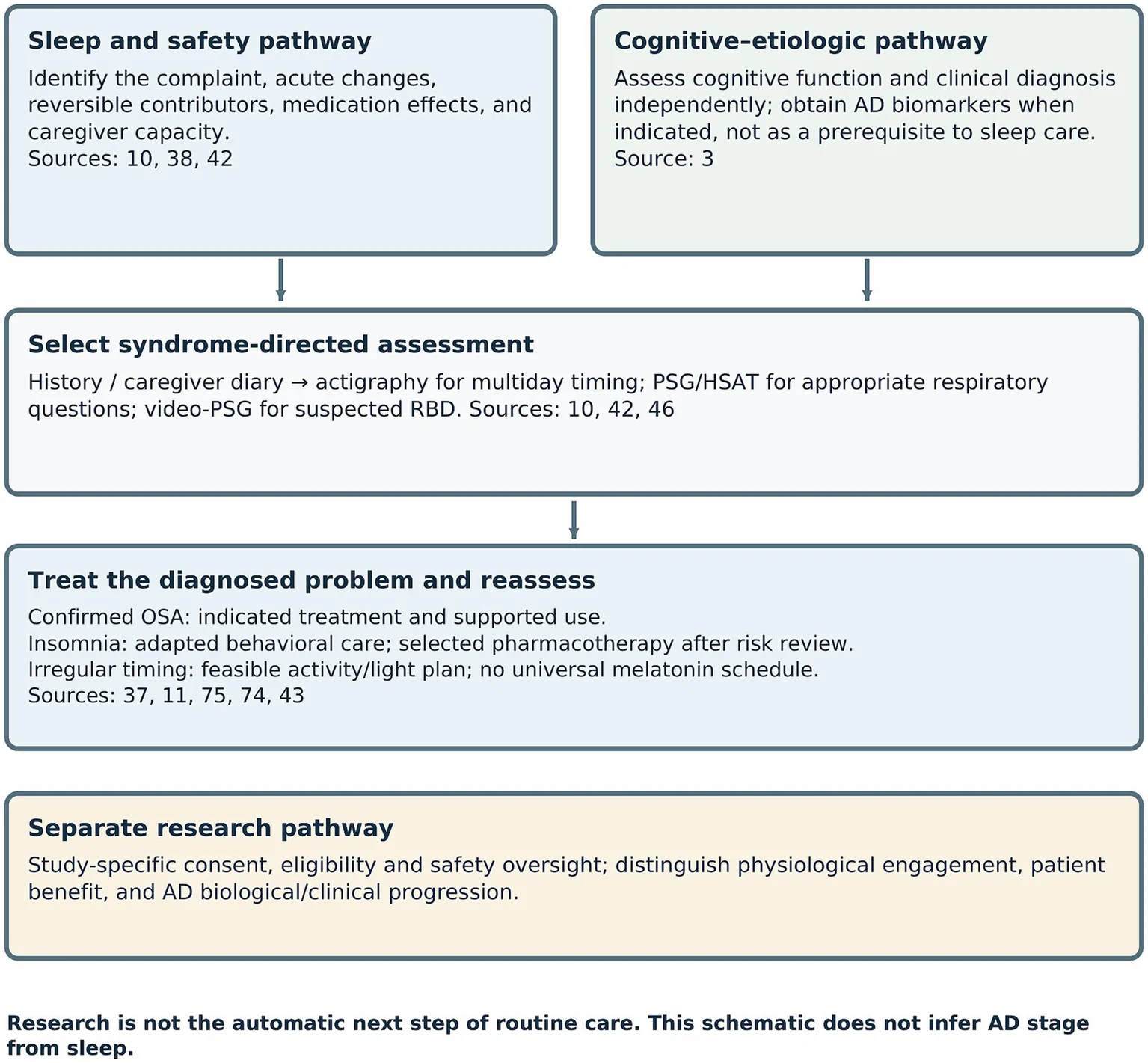
Symptom-directed sleep care and cognitive-etiologic assessment proceed in parallel. History, reversible contributors, medication exposure and caregiver feasibility guide syndrome-specific testing and follow-up (10, 38, 42, 46). Treatment follows established indications, with dementia-adapted behavioral care and diagnosis-specific circadian recommendations (11, 37, 43, 74, 75). Biomarker-defined study eligibility and experimental outcomes are reserved for the relevant research question. AD status is not inferred from sleep and is not a universal prerequisite for sleep care. AD, Alzheimer’s disease; HSAT, home sleep apnea testing; OSA, obstructive sleep apnea; PSG, polysomnography; RBD, REM sleep behavior disorder.

Follow-up should repeat the outcome that motivated treatment. Insomnia care should assess continuity and daytime impact; PAP should assess respiratory control, actual use, and sleep-related function; timing interventions should assess the 24-h pattern; caregiver outcomes should be measured separately. Improvement in a questionnaire cannot establish improved oscillatory physiology, and a physiological change need not reduce care burden. Table 4 contrasts selected intervention studies and guidelines, while Table 5 matches clinical and research questions to endpoints.

**Table 4:** Selected intervention contrasts: the population, comparator, and endpoint determine the claim.

Intervention / source population	Design / exposure	Principal result	Clinical interpretation
Ancoli-Israel: CPAP in clinical AD + OSA (70)	52 participants; therapeutic versus sham CPAP; initial randomized comparison at 3 weeks.	No significant randomized cognitive advantage; pooled therapeutic pre–post results were exploratory.	Treat confirmed OSA; pooled improvement does not establish AD prevention.
Richards: CPAP adherence in MCI + OSA (72)	54 one-year completers; ≥4 versus <4 h/night; nonrandomized adherence groups. Primary cognitive outcome: memory.	Secondary Digit Symbol processing-speed outcome: adjusted estimate 1.68 (95% CI 0.73–2.62), favoring adherers.	An adherence association on a secondary endpoint; not a randomized dose effect, primary memory benefit, or disease modification.
Xu: cognitively normal OSA (73)	148 adults; CPAP plus supportive care versus supportive care; 6 months.	MoCA difference −0.04 (95% CI − 0.72 to 0.65); some MRI endpoints differed.	Non-AD counterexample: imaging response does not establish cognitive benefit or amyloid/tau modification.
Herring: suvorexant for probable-AD insomnia (75)	285 participants; placebo-controlled; 4 weeks.	TST difference 28 min (95% CI 11–45); somnolence 4.2% vs. 1.4%; falls 3/142 vs. 0/143.	Short-term symptomatic efficacy in this population; assess next-day function, falls, and interactions.
Lucey: acute suvorexant physiology (76)	38 cognitively unimpaired middle-aged adults; placebo comparison; serial CSF sampling.	Acute Aβ and selected site-specific tau-phosphorylation changes.	Human target engagement, not a prevention or longitudinal AD treatment trial.
McCurry: NITE-AD (74)	36 AD patient–caregiver dyads; multicomponent program versus control; 2-month assessment and 6-month follow-up.	Improved activity-derived nocturnal outcomes following caregiver-supported hygiene, walking, and light.	Direct dementia-care context; the combined program does not isolate each component or equal standard CBT-I.
Insomnia / circadian guidelines (11, 37)	Adult insomnia guidance and disorder-specific circadian recommendations.	CBT-I requires cognitive/caregiver adaptation; AASM suggests against melatonin for dementia-associated ISWRD.	Separate guideline recommendation from extrapolation; no blanket light/melatonin schedule.
Richards: gabapentin enacarbil in AD + RLS/agitation (48)	147 participants; randomized placebo-controlled syndrome-selected trial.	Agitation and actigraphic sleep improved; adverse events and falls require attention.	Do not generalize to unexplained nocturnal agitation or all insomnia.
Acoustic slow-wave stimulation (82–85)	Small MCI/AD studies and mixed healthy/impaired older samples; varied within-person designs. Meta-analysis (85) includes healthy participants only.	SWA can increase during stimulation. The healthy-participant pooled memory effect was not significant; AD cognitive efficacy remains unestablished.	Research intervention; physiological engagement and sustained clinical efficacy are separate endpoints.

Selection rationale: these examples contrast direct AD trials, nonrandomized MCI adherence findings, a non-AD randomized counterexample, guideline-based care, and experimental target engagement. They are not all eligible studies or a formal certainty ranking. AASM, American Academy of Sleep Medicine; AD, Alzheimer’s disease; CBT-I, cognitive behavioral therapy for insomnia; CI, confidence interval; CPAP, continuous positive airway pressure; CSF, cerebrospinal fluid; ISWRD, irregular sleep–wake rhythm disorder; MCI, mild cognitive impairment; MoCA, Montreal Cognitive Assessment; MRI, magnetic resonance imaging; OSA, obstructive sleep apnea; PSG, polysomnography; RLS, restless legs syndrome; SWA, slow-wave activity; TST, total sleep time.

**Table 5:** Match a clinical or research decision to its population, measure, and endpoint.

Decision / context	Evidence required	Appropriate endpoint	Inference to avoid
Treat a diagnosed sleep syndrome at any cognitive stage	Applicable diagnostic criteria, syndrome-specific treatment evidence, individual feasibility and harms.	Symptoms, continuity, respiratory control, daytime function, safety and tolerability.	Treatment success proves an AD-specific mechanism (37, 42, 43, 75)
Interpret a rest–activity association	Cohort, device, metric and unit, outcome definition, follow-up, confounding and shared samples.	The actual incident clinical endpoint; external prediction performance only when evaluated.	Behavioral regularity universally prevents AD; an OR equals a per-SD HR (12–16)
Interpret biomarker-linked sleep physiology	Separate clinical diagnosis, eligibility biomarkers, measured biomarker outcomes and assay modality.	Specific PET/CSF/plasma outcome and sleep feature, with temporal and subgroup context.	An amyloid result automatically supplies tau positivity, or a sleep feature assigns AD stage (3, 19, 20, 23, 29)
Test a sleep-targeting intervention	Delivered exposure, adequate comparator, prespecified physiological and clinical outcomes.	Target engagement, patient-relevant benefit, and AD biological/clinical change measured separately.	Larger slow waves or changed CSF concentrations prove disease modification (76, 82, 83)
Evaluate dementia care	Clinical diagnosis and setting; patient capability, caregiver resources, medication and implementation burden.	Patient sleep/daytime function, caregiver burden, falls and treatment harms.	Mixed-dementia effectiveness establishes an AD-specific pathway (35, 48, 74, 77)
Study fluid transport	Species, tracer route, compartment, vigilance state and measurement interval.	Influx, redistribution, retention, clearance or coupled oscillation, as actually measured.	Human CSF oscillations directly quantify long-term net amyloid clearance (61–67)

An interpretive structure, not a validated algorithm or new evidence-grading instrument. AD, Alzheimer’s disease; CSF, cerebrospinal fluid; HR, hazard ratio; OR, odds ratio; PET, positron emission tomography; SD, standard deviation.

### 6.2 PAP: separate the treatment indication from cognitive efficacy

The Ancoli-Israel trial enrolled 52 patients with mild-to-moderate AD and OSA. Therapeutic versus placebo continuous positive airway pressure (CPAP) at 3 weeks did not show a significant cognitive advantage; pooled before–after therapeutic analyses suggested improvement but were exploratory (70). Observational associations between PAP and lower dementia incidence remain susceptible to access-to-care and healthy-adherer effects (71). The systematic review by Shieu and colleagues describes heterogeneous designs and cognitive populations rather than definitive prevention evidence (44).

In MEMORIES 1, 54 one-year completers with MCI and OSA were compared by average CPAP use of at least versus less than 4 hours/night (72). Memory was the primary cognitive outcome; the reported adjusted difference of 1.68 (95% CI 0.73–2.62) favored adherers on the secondary Digit Symbol processing-speed outcome. This nonrandomized adherence comparison does not establish a causal dose–response, a primary memory benefit, or AD modification. The 2025 Xu trial in 148 cognitively normal adults with OSA offers a brief counterexample: the six-month Montreal Cognitive Assessment (MoCA) difference was −0.04 (95% CI − 0.72 to 0.65), despite differences in MRI outcomes (73). Its non-AD population and non-amyloid/tau imaging measures limit its relevance to AD treatment.

### 6.3 Insomnia care and orexin antagonism

Behavioral insomnia treatment in adults should be adapted to cognitive capacity and caregiver resources (37). MCI may allow participation in structured therapy; advanced dementia often requires simplified routines and caregiver-led implementation. A direct dementia example is NITE-AD: 36 community-dwelling AD patient–caregiver dyads were randomized to a multicomponent program or control, with activity-derived sleep assessments after 2 months and at 6 months (74). The intervention combined sleep hygiene, walking, light, and caregiver behavior management; improved nocturnal outcomes cannot isolate the effect of any one component. Sleep opportunity, daytime activity, and medication timing should be reviewed before sedative escalation. Aggressive sleep restriction is not applied mechanically to frail patients with substantial falls risk or limited ability to follow instructions.

Suvorexant has randomized evidence for short-term insomnia treatment in mild-to-moderate probable AD dementia. Among 285 randomized participants, the four-week between-group gain in PSG total sleep time was 28 min (95% CI 11–45) (75). Somnolence was reported in 4.2% with suvorexant versus 1.4% with placebo; falls occurred in three participants (2.1%) versus none. These observations support a drug- and population-specific sleep benefit alongside explicit assessment of next-day alertness, falls, interactions, and other CNS depressants. They do not establish class-wide efficacy, long-term functional benefit, or slowed AD progression (38, 75).

A separate 38-participant study in cognitively unimpaired middle-aged adults found acute CSF amyloid and selected tau-phosphorylation changes after suvorexant (76). That result supplies a pharmacodynamic hypothesis, not evidence that treating AD insomnia changes the disease course. The symptomatic trial and the biomarker experiment should therefore be presented side by side, not joined into a single therapeutic claim. Clinical prescribing follows the insomnia indication; disease-modifying use requires dedicated trials with appropriate AD ascertainment and longitudinal endpoints.

### 6.4 Circadian strengthening, light, and melatonin

Regular daytime activity, adequate daytime light exposure, and a consistent care routine address modifiable contributors to an irregular 24-h pattern. Dementia lighting studies demonstrate feasibility and potential benefit, while phase-tailored work emphasizes that timing matters (77, 78). Light, activity, and caregiver support are often delivered together, making the effective component difficult to isolate. Variation in visual function, exposure intensity, timing, season, setting, and adherence also limits transportability. Low burden does not mean that every protocol has established efficacy for cognition or nighttime agitation.

Melatonin requires a diagnosis-specific interpretation. The American Academy of Sleep Medicine (AASM) guideline suggests light therapy for irregular sleep–wake rhythm disorder in older adults with dementia and suggests against melatonin in this setting; it also recommends against sleep-promoting medication for this specific disorder (11). A recent meta-analysis in heterogeneous cognitive-impairment populations reported improvement on the Mini-Mental State Examination (MMSE) but not the Alzheimer’s Disease Assessment Scale–Cognitive Subscale (ADAS-Cog) (79). Such scale-dependent results do not override disorder-specific recommendations. Figure 6 separates routine activity/light support from selected pharmacological treatment. Daytime light/activity support, a phase-directed treatment, ordinary insomnia care, and management of nonspecific evening agitation remain distinct clinical decisions.

### 6.5 Experimental slow-wave enhancement: physiological response is not clinical benefit

Acoustic stimulation in healthy adults provides proof of principle for modifying slow oscillations; it is not itself evidence of efficacy in AD (80, 81). In the randomized crossover study by Papalambros et al. (82), nine adults with amnestic MCI showed increased SWA during stimulated intervals, but whole-night NREM SWA and memory performance did not differ significantly between stimulation and sham. In a separate open-label at-home study, 15 patients with AD completed baseline nights followed by randomly assigned stimulation or sham nights. The study demonstrated SWA and SWS enhancement, but its small sample and physiological endpoints do not establish durable cognitive benefit (83). These findings distinguish a measurable response to stimulation from an effective clinical treatment.

Zeller et al. (84) studied 18 cognitively healthy older adults and 16 older adults with screening-defined cognitive impairment, relating multi-night physiological responses to memory and plasma amyloid measures. Wunderlin’s et al. (85) meta-analysis included 10 studies with 11 experiments and 177 healthy participants; the overall memory effect was small and not statistically significant (Hedges’ g = 0.25, *p* = 0.07), despite selected subgroup signals. These healthy-participant findings are not an estimate of efficacy in AD. Small samples, differing cognitive definitions, stimulus delivery, and responder analyses limit generalization. Further sham-controlled work should prespecify clinical outcomes and tolerability. Evidence from acoustic stimulation does not establish the efficacy of electrical or pharmacological approaches to oscillatory modulation.

## 7 Discussion

The evidence supports a clinically useful separation: sleep disorders can be actionable without being specific to AD, whereas an AD-associated physiological signal may have no established clinical intervention. This separation avoids two opposite errors. Treating all sleep disturbance as inevitable neurodegeneration can miss OSA, insomnia, medication effects, or RLS. Treating every association with amyloid or cognition as an indication for disease-modifying sleep therapy overstates the available evidence. The purpose of stage-aware reasoning is to match the patient’s problem and capacity to an appropriate assessment and outcome, not to infer a disease stage from sleep.

Several comparisons make this conclusion concrete. Activity-derived rhythms predict different cognitive endpoints across cohorts but are also shaped by movement and environment. PSG and quantitative EEG provide greater physiological detail, yet age-related and MCI-associated findings overlap. PAP and suvorexant have different evidence bases and different tested outcomes. A favorable MRI or CSF response does not resolve a null cognitive result, and a successful insomnia intervention need not modify amyloid or tau to be valuable. These contrasts give the review its practical contribution: they identify which conclusion follows from each study design rather than placing all sleep–AD evidence on one scale.

For clinicians, the immediate implication is an indication-based assessment with explicit follow-up goals. A memory service should know whether it is investigating insomnia, breathing disturbance, abnormal behavior, or an irregular daily pattern before choosing a test. Changes in medications, caregiver availability, or physical health should be documented alongside recordings. Referral to sleep medicine is particularly relevant for suspected complex sleep-disordered breathing, injurious parasomnia, uncertain movement-related symptoms, or persistent impairment despite initial management. None of these actions requires portraying sleep measures as standalone diagnostic biomarkers of AD (10, 42, 46).

For research, AD ascertainment and endpoint selection should be built into the design rather than repaired in the discussion. Biomarker-supported early-AD studies can test whether a specified intervention changes its intended sleep target and subsequently modifies independently measured pathology. Later-stage studies may appropriately prioritize continuity, daytime function, distress, falls, or caregiver burden. All-cause dementia cohorts can address care effectiveness but should not be relabeled AD cohorts for mechanistic interpretation. Preplanned stratification and repeated measurements are preferable to *post hoc* assertions that a heterogeneous group represents the entire AD continuum.

The purposive search limits the conclusions in several ways. Retrieval completeness and the impact of omitted studies cannot be quantified, and study-level bias was not assessed using a formal instrument. The treatment table illustrates selected contrasts rather than estimating a pooled effect or overall certainty across all eligible trials. Clinical application draws on applicable guidelines and bounded study results; adaptation to advanced dementia sometimes rests on clinical reasoning. Biomarker cohorts are selected, treatment trials differ in endpoints and follow-up, and overlapping cohorts are not independent replications. The review therefore provides a clinical interpretation of identified evidence, with explicit source populations and negative findings, rather than a comprehensive efficacy ranking.

## 8 Conclusion

Sleep and circadian care in AD should begin with the clinical syndrome, not an assumed molecular mechanism. History and caregiver observations, longitudinal activity monitoring, and targeted PSG answer complementary questions. Confirmed OSA, clinically significant insomnia, phase disturbance, and selected parasomnias or limb symptoms warrant indication-specific care with attention to cognition, safety, and caregiver feasibility. Current evidence supports separating symptomatic benefit from physiological target engagement and both from disease modification. A more informative research agenda will define AD etiology independently, specify the sleep metric and comparator, retain negative outcomes, and measure whether biological effects translate into durable patient-relevant benefit.
