# Supplementary material for: Sleep and circadian disturbances in Alzheimer’s disease: clinical assessment, treatable syndromes, and limits of biomarker inference
Source: Front Neurol. 2026 Oct 28;17:1998439. doi: 10.3389/fneur.2026.1998439 (PMC13752404; doi:10.3389/fneur.2026.1998439)
Supplement: Supplementary file 1 [file Table_1.docx]

Supplementary Material

# Table S1. Narrative search, source selection, and reporting.

| **Item** | **Reporting** |
| --- | --- |
| Review design | Purposive narrative review. No prospective registration, systematic duplicate screening, new meta-analysis, or formal study-level risk-of-bias scoring was performed. |
| Historical search | The submitted manuscript reported PubMed/MEDLINE and citation searches through 17 July 2026, followed by focused record verification on 14 September 2026. An executable historical screening log is not reconstructed. |
| Current update | 4 October 2026: targeted web searches of PubMed-indexed bibliographic/abstract records and original publisher reports; direct DOI/PMID retrieval and cited-reference follow-up. This is not an exhaustive PubMed database search. |
| Concepts | Sleep and AD; circadian physiology versus rest–activity behavior; PAP/cognition and treatment exposure; medication effects; daytime sleepiness; AD assays; orexin and sleep-related fluid transport. |
| Restrictions | English-language search terms. No date or study-design filter for source verification; 2025–2026 records and reviewer-specified studies were prioritized where relevant. No claim of exhaustive non-English retrieval. |
| Inclusion rationale | Sources answering a clinical question, supporting a displayed/numerical statement, distinguishing a population or biomarker context, or materially challenging an interpretation. Guidelines inform indications; primary reports inform the actual comparator and result. |
| Negative evidence | Prespecified null randomized endpoints, non-significant multiplicity-adjusted findings, discordant tracer experiments, harms, and nonrandomized adherence comparisons were retained. Positive within-group results do not replace randomized comparisons. |
| Intervention examples | Table 4 deliberately contrasts AD/OSA randomized evidence, MCI adherence associations, a non-AD counterexample, AD insomnia treatment, dementia-adapted behavior, guidelines, syndrome-selected therapy, and experimental physiology. It is not a census or treatment ranking. |
| What is counted | The bibliography and Table S2 describe retained sources. They are not identification, screening, exclusion, or eligible-study counts. Omitted evidence and retrieval completeness cannot be quantified. |
| Appraisal | Population, ascertainment, assay, comparator, endpoint and major limitation are described. No evidence-maturity scale, GRADE assessment, inter-rater statistic or validated decision score is claimed. |
| Source availability | Publisher full text or source PDF was consulted where accessible; PubMed/publisher abstracts supplied bounded summaries when full text was unavailable. Assay thresholds or numerical results are not inferred from titles or reconstructed from P values. |

## Retained targeted queries executed for this revision

Each entry below is an actual targeted web query used on 4 October 2026. Search output was used to locate the original record, not treated as a comprehensive screening sample. Source URLs in Table S2 identify the underlying publications. Multiple query results can refer to the same study.

| **Executed query** | **Purpose** |
| --- | --- |
| "34753629" donepezil | Donepezil sleep-architecture review: exact title, authors, REM and continuity outcomes. |
| "41626291" "Interdaily" | ARIC variability study: distinguish median absolute deviations from conventional IV. |
| "42096659" | Down-syndrome rest–activity study: population, cognitive outcomes, and multiplicity. |
| "35523585" | PAP/cognitive systematic review identified by Reviewer 2. |
| "10.1513/AnnalsATS.202205-380ST" authors | ATS workshop: cognition, OSA phenotype and exposure context. |
| "29532057" | Sleepiness and longitudinal amyloid PET, not a treatment trial. |
| "10.1111/j.1532-5415.2005.53252.x" McCurry sleep 36 | NITE-AD trial: clinical population and combined components. |
| "10.5664/jcsm.7640" Patil 2019 335 | PAP guideline and bibliographic verification. |
| "10.1111/j.1532-5415.2010.02733.x" title | Mixed-dementia caregiver-reported prevalence. |
| "10.1093/brain/awaa443" 24 sleep | Human intrathecal-tracer evidence and clinical sample. |
| "10.1186/s13195-026-02088-3" | Genetic susceptibility/rest–activity analysis and overlap with UK Biobank. |
| "10.5665/sleep.5846" | Correct original first author and title for CSF orexin–p-tau study. |
| "CPAP Adherence May Slow" "primary" "Digit" | Verify primary versus secondary cognitive endpoints and the processing-speed estimate. |
| "10.1002/alz.12035" "falls" | Verify somnolence and fall counts alongside four-week sleep benefit. |
| "Circadian Rest-Activity Pattern Changes" "189" "PiB" | Specify pathological assay, analysis subsets, and actigraphy result. |
| "Reduced non-rapid eye movement sleep is associated with tau pathology" "119" | Verify cohort and the distinct PET/CSF modalities used with EEG. |
| "Amyloid burden is associated with self-reported sleep" "98" | Retain positive and null questionnaire outcomes and amyloid-PET ligand. |
| "10.1002/trc2.70219" | Verify metric definitions and disclose abstract/full-text sample-count discrepancy. |
| "10.1016/j.neuroscience.2021.11.042" | Original author list for Duncan’s mouse fragmentation study. |
| "10.1126/scitranslmed.ade6285" | Original author list for Parhizkar’s TREM2 study. |
| "aba5187" | Published Lucey correction: CSF-specific analysis denominator. |
| "10.1093/sleep/zsaa296" | Healthy-participant eligibility and the overall acoustic-stimulation memory result. |
| "10.1007/s11357-024-01195-z" | Healthy and screening-defined impaired groups in Zeller’s study. |

# Table S2. Selected study-to-claim evidence map.

This map supports the clinical contrasts and selected numerical or biomarker statements in the manuscript. It is a purposive source register, not all studies eligible for a systematic review. Reference numbers correspond to the main manuscript. Clinical diagnosis, AD-defining eligibility, and a biomarker measured as an outcome are kept separate; absent assay details are not retrospectively assigned.

## Reference 12: Winer JR, Lok R, Weed L, He Z, Poston KL, Mormino EC, et al. Impaired 24-h activity patterns are associated with an increased risk of Alzheimer’s disease, Parkinson’s disease, and cognitive decline. Alzheimers Res Ther

| **Field** | **Source-specific context** |
| --- | --- |
| Population / ascertainment | 82,829 UK Biobank adults; 187 record-defined AD events. No uniform pathological adjudication. |
| Measurement / assay | Wrist activity: IS, amplitude, mesor, M10; hospital/primary-care outcome records. |
| Design / comparator | Prospective; mean 6.8 years; per-SD Cox estimates. |
| Reported finding | IS HR 1.25 (1.05–1.48); amplitude .79 (.65–.96); mesor abstract .77 (.59–.998), main table .78; M10 .75 (.61–.94). |
| Reason for inclusion / limit | Quantitative Figure 2 example; illustrates metric-specific direction and source discrepancy. |
| Original source | https://doi.org/10.1186/s13195-024-01411-0 |

## Reference 13: Posner AB, Tranah GJ, Blackwell T, Yaffe K, Ancoli-Israel S, Redline S, et al. Predicting incident dementia and mild cognitive impairment in older women with nonparametric analysis of circadian activity rhythms in the Study of Osteoporotic Fractures. Sleep

| **Field** | **Source-specific context** |
| --- | --- |
| Population / ascertainment | 1,232 older women in SOF; incident dementia distinct from MCI. |
| Measurement / assay | Nonparametric wrist rhythms: L5 midpoint and RA. |
| Design / comparator | Prospective categorical logistic estimates; approximately 5 years. |
| Reported finding | Earlier L5 OR 1.66 (1.08–2.55); low RA OR 1.96 (1.14–3.35), for dementia. |
| Reason for inclusion / limit | Figure 2 OR block; not the same contrast as standardized HR; shared SOF source with Tranah. |
| Original source | https://doi.org/10.1093/sleep/zsab119 |

## Reference 9: Tranah GJ, Blackwell T, Stone KL, Ancoli-Israel S, Paudel ML, Ensrud KE, et al. Circadian activity rhythms and risk of incident dementia and mild cognitive impairment in older women. Ann Neurol

| **Field** | **Source-specific context** |
| --- | --- |
| Population / ascertainment | Older women in SOF; combined MCI/dementia endpoint. |
| Measurement / assay | Actigraphic parametric rhythm measures. |
| Design / comparator | Prospective observational cohort. |
| Reported finding | Amplitude and acrophase related to combined incident cognitive endpoint. |
| Reason for inclusion / limit | Foundational context; overlap with Posner prevents independent-cohort counting. |
| Original source | https://doi.org/10.1002/ana.22468 |

## Reference 14: Wang W, Wanigatunga AA, Etzkorn LH, Rabinowitz JA, Palta P, Pike JR, et al. Association between circadian rest-activity rhythms and incident dementia in older adults: The Atherosclerosis Risk in Communities Study. Neurology

| **Field** | **Source-specific context** |
| --- | --- |
| Population / ascertainment | 2,183 older ARIC participants; incident dementia, not biomarker-defined AD. |
| Measurement / assay | Chest-worn ambulatory-ECG accelerometry; RA and IV. |
| Design / comparator | Prospective, median approximately 3 years. |
| Reported finding | Lower RA and higher IV associated with incident dementia. |
| Reason for inclusion / limit | Recent metric/outcome contrast; not an endogenous phase assay. |
| Original source | https://doi.org/10.1212/WNL.0000000000214513 |

## Reference 15: Chamberlin KW, Etzkorn LH, Davoudi A, Wang W, Dougherty RJ, Palta P, et al. Interdaily variability of rest–activity patterns and risk of dementia in older adults: the ARIC Neurocognitive Study. Alzheimers Dement (N Y)

| **Field** | **Source-specific context** |
| --- | --- |
| Population / ascertainment | ARIC older adults; the abstract reports n=2,332, whereas the full-text cohort flow and main tables report n=2,335. Mean age approximately 79 years; 319 incident dementia events. |
| Measurement / assay | ZioXT activity; median absolute deviation of daily/M10/L5 volume and timing. |
| Design / comparator | Prospective; median 5.2 years. |
| Reported finding | Daytime-volume variability and timing variability had different directions; L5 timing attenuated with activity adjustment. |
| Reason for inclusion / limit | Separates day-to-day activity-volume variability from timing variability and conventional IV. The source abstract/full-text sample-count discrepancy is retained rather than silently reconciled. |
| Original source | https://doi.org/10.1002/trc2.70219 |

## Reference 16: Chung PA, Fleming VL, Reid KJ, Plante DT, Hom CL, Ances BM, et al. Circadian rest-activity rhythms, cognition, and Alzheimer disease dementia in adults with Down syndrome. Neurology

| **Field** | **Source-specific context** |
| --- | --- |
| Population / ascertainment | 115 adults with Down syndrome; clinical cognitive-status comparison. |
| Measurement / assay | Wrist actigraphy and cognitive assessments; ≥4 valid recording days. |
| Design / comparator | Cross-sectional; not sporadic incident-AD replication. |
| Reported finding | Higher IS associated with better cognition; IV–MCI/dementia association did not remain significant after FDR. |
| Reason for inclusion / limit | Down-syndrome comparator; illustrates population and multiplicity boundaries. |
| Original source | https://doi.org/10.1212/WNL.0000000000214938 |

## Reference 17: Yang S, Xiao X, Yang Y, Cai Y, Zou Y, Tang Y, et al. Alzheimer's disease polygenic risk, *APOE* ε4 dose, and accelerometer-derived circadian rest–activity rhythms in relation to incident dementia and Alzheimer's disease in UK Biobank: a prospective cohort study. Alzheimers Res Ther

| **Field** | **Source-specific context** |
| --- | --- |
| Population / ascertainment | 80,378 UK Biobank participants in longitudinal analysis; 949 dementia and 371 AD events. |
| Measurement / assay | Accelerometer rhythms, AD PRS and *APOE* ε4 dose; record-based outcomes. |
| Design / comparator | Prospective genetic-context analysis. |
| Reported finding | RA associations differed by outcome/activity adjustment; tested genetic interactions were not significant. |
| Reason for inclusion / limit | Relevant 2026 update; genotype is susceptibility, not pathology; overlaps UK Biobank source. |
| Original source | https://doi.org/10.1186/s13195-026-02088-3 |

## Reference 19: Musiek ES, Bhimasani M, Zangrilli MA, Morris JC, Holtzman DM, Ju Y-ES. Circadian rest-activity pattern changes in aging and preclinical Alzheimer disease. JAMA Neurol

| **Field** | **Source-specific context** |
| --- | --- |
| Population / ascertainment | 189 cognitively normal adults; mean age 66.6 years, 64% women. Biomarker subsets differ: 142 had PiB PET, including 26 PiB-positive participants. |
| Measurement / assay | Actigraphy; [11C]Pittsburgh compound B amyloid PET and CSF phosphorylated-tau/Aβ42 ratio in available subsets. PET classification and continuous CSF ratio are distinct analyses. |
| Design / comparator | Observational biomarker-context analysis. |
| Reported finding | Age/sex-adjusted IV was higher with PiB positivity (0.875 vs 0.804; P=.05) and increasing CSF p-tau/Aβ42 (β=.231; P=.008); associations do not establish intrinsic specificity. |
| Reason for inclusion / limit | AD-linked biomarker analysis; no inference that every participant is A+T+ or that actigraphy diagnoses AD. |
| Original source | https://doi.org/10.1001/jamaneurol.2017.4719 |

## Reference 20: Ju Y-ES, McLeland JS, Toedebusch CD, Xiong C, Fagan AM, Duntley SP, et al. Sleep quality and preclinical Alzheimer disease. JAMA Neurol

| **Field** | **Source-specific context** |
| --- | --- |
| Population / ascertainment | 142 evaluable cognitively normal participants; 32 CSF-amyloid-positive. |
| Measurement / assay | Two-week actigraphy plus diaries; amyloid group defined by CSF Aβ42 ≤500 pg/mL in the source assay, not by PSG or mandatory tau positivity. |
| Design / comparator | Observational group comparison; biomarker and activity records not necessarily simultaneous. |
| Reported finding | Sleep efficiency 80.4% versus 83.7%; adjusted P=.04; no significant total-sleep-time difference. |
| Reason for inclusion / limit | Activity-derived sleep measure; amyloid definition is not tau confirmation. |
| Original source | https://doi.org/10.1001/jamaneurol.2013.2334 |

## Reference 22: Mander BA, Marks SM, Vogel JW, Rao V, Lu B, Saletin JM, et al. β-Amyloid disrupts human NREM slow waves and related hippocampus-dependent memory consolidation. Nat Neurosci

| **Field** | **Source-specific context** |
| --- | --- |
| Population / ascertainment | Older adults characterized with amyloid PET and sleep/memory measures. |
| Measurement / assay | Amyloid PET; NREM slow waves and hippocampal-dependent memory. |
| Design / comparator | Human observational physiology. |
| Reported finding | Amyloid burden related to slow-wave/memory measures. |
| Reason for inclusion / limit | Mechanistic human association; not a sleep-treatment trial or a validated diagnostic threshold. |
| Original source | https://doi.org/10.1038/nn.4035 |

## Reference 23: Lucey BP, McCullough A, Landsness EC, Toedebusch CD, McLeland JS, Zaza AM, et al. Reduced non-rapid eye movement sleep is associated with tau pathology in early Alzheimer’s disease. Sci Transl Med

| **Field** | **Source-specific context** |
| --- | --- |
| Population / ascertainment | 119 recruited adults older than 60 years, predominantly cognitively normal or very mildly impaired. PET analyses included 38 participants; the 2020 erratum corrects the CSF-specific models to 23 participants. These analysis denominators must not be replaced by the overall cohort size. |
| Measurement / assay | Home single-channel EEG for up to six nights; [18F]AV-45 amyloid PET, [18F]AV-1451 tau PET, and CSF Aβ42, total tau and phosphorylated tau in the relevant subsets. |
| Design / comparator | Observational biomarker-linked sleep analysis. |
| Reported finding | Lower-frequency NREM SWA, particularly 1–2 Hz, showed inverse associations with pathology, more pronounced for tau than amyloid. Broad sleep-log/actigraphic measures were not interchangeable with SWA. |
| Reason for inclusion / limit | Assay- and frequency-specific evidence. The published correction changes the CSF-analysis participant count in the text, Tables 4–5 and Table S5 to 23; consult the corrected report rather than the uncorrected repository manuscript. |
| Original source | https://doi.org/10.1126/scitranslmed.aau6550; correction: https://doi.org/10.1126/scitranslmed.aba5187 |

## Reference 25: Ladenbauer J, Ladenbauer J, Külzow N, Flöel A. Memory-relevant nap sleep physiology in healthy and pathological aging. Sleep

| **Field** | **Source-specific context** |
| --- | --- |
| Population / ascertainment | Young adults, healthy older adults and clinical MCI; no uniform AD pathology requirement. |
| Measurement / assay | 90-minute nap physiology, SO–spindle coupling and memory. |
| Design / comparator | Cross-sectional/nap comparison. |
| Reported finding | Several older-control versus MCI comparisons were not significant. |
| Reason for inclusion / limit | Negative comparison retained; does not establish equivalence or AD-specific causation. |
| Original source | https://doi.org/10.1093/sleep/zsab002 |

## Reference 28: André C, Martineau-Dussault M-È, Daneault V, Blais H, Frenette S, Lorrain D, et al. REM sleep is associated with the volume of the cholinergic basal forebrain in aMCI individuals. Alzheimers Res Ther

| **Field** | **Source-specific context** |
| --- | --- |
| Population / ascertainment | Amnestic MCI, without uniform AD etiologic confirmation. |
| Measurement / assay | REM sleep and MRI basal-forebrain volume. |
| Design / comparator | Observational imaging association. |
| Reported finding | REM associated with cholinergic basal-forebrain volume. |
| Reason for inclusion / limit | MRI volume is not an amyloid/tau eligibility criterion. |
| Original source | https://doi.org/10.1186/s13195-023-01265-y |

## Reference 29: Jin J, Chen J, Cavaillès C, Yaffe K, Winer J, Stankeviciute L, et al. Association of rapid eye movement sleep latency with multimodal biomarkers of Alzheimer’s disease. Alzheimers Dement

| **Field** | **Source-specific context** |
| --- | --- |
| Population / ascertainment | 128 clinical AD/MCI/normal-cognition participants. |
| Measurement / assay | PSG; 18F-florbetapir amyloid PET; plasma p-tau181, NfL and BDNF. |
| Design / comparator | Cross-sectional multimodal analysis. |
| Reported finding | Longer REM latency associated with selected amyloid/plasma p-tau181 results; SWS results were not robust in adjusted/multiplicity context. |
| Reason for inclusion / limit | Specifies plasma tau rather than tau PET; removes generalized positive/negative heat-map claims. |
| Original source | https://doi.org/10.1002/alz.14495 |

## Reference 30: Spira AP, Gamaldo AA, An Y, Wu MN, Simonsick EM, Bilgel M, et al. Self-reported sleep and β-amyloid deposition in community-dwelling older adults. JAMA Neurol

| **Field** | **Source-specific context** |
| --- | --- |
| Population / ascertainment | 70 community-dwelling older adults. |
| Measurement / assay | Self-reported sleep; [11C]Pittsburgh compound B PET mean cortical and precuneus distribution-volume ratios. PET is an outcome, not proof of symptomatic AD in the cohort. |
| Design / comparator | Cross-sectional observational analysis. |
| Reported finding | Subjective sleep related to amyloid deposition. |
| Reason for inclusion / limit | Self-report/PET association; cannot be transferred to PSG stages or a tau threshold. |
| Original source | https://doi.org/10.1001/jamaneurol.2013.4258 |

## Reference 31: Sprecher KE, Bendlin BB, Racine AM, Okonkwo OC, Christian BT, Koscik RL, et al. Amyloid burden is associated with self-reported sleep in nondemented late middle-aged adults. Neurobiol Aging

| **Field** | **Source-specific context** |
| --- | --- |
| Population / ascertainment | 98 cognitively healthy late-middle-aged WRAP participants; mean age 62.4 years at PET. Mean interval from PET to sleep questionnaire approximately 0.69 years. |
| Measurement / assay | [11C]Pittsburgh compound B amyloid PET; Medical Outcomes Study Sleep Scale and Epworth Sleepiness Scale. |
| Design / comparator | Observational biomarker comparison. |
| Reported finding | Selected sleep-quality and MOS somnolence scores were associated with regional amyloid; reported sleep amount, sleep-disordered-breathing symptoms, sleep-onset difficulty, and Epworth scores were not. |
| Reason for inclusion / limit | Retains positive and null measures within the same study. Sleep quality, sleep duration and sleepiness instruments cannot be treated as interchangeable. |
| Original source | https://doi.org/10.1016/j.neurobiolaging.2015.05.004 |

## Reference 32: Ooms S, Overeem S, Besse K, Olde Rikkert M, Verbeek M, Claassen JAHR. Effect of 1 night of total sleep deprivation on cerebrospinal fluid β-amyloid 42 in healthy middle-aged men: A randomized clinical trial. JAMA Neurol

| **Field** | **Source-specific context** |
| --- | --- |
| Population / ascertainment | 26 healthy middle-aged men. |
| Measurement / assay | One-night sleep manipulation; CSF Aβ42. |
| Design / comparator | Randomized acute sleep-deprivation experiment. |
| Reported finding | Deprivation altered the overnight CSF Aβ42 pattern. |
| Reason for inclusion / limit | Human acute physiological response, not chronic insomnia treatment in AD. |
| Original source | https://doi.org/10.1001/jamaneurol.2014.1173 |

## Reference 33: Shokri-Kojori E, Wang GJ, Wiers CE, Demiral SB, Guo M, Kim SW, et al. β-Amyloid accumulation in the human brain after one night of sleep deprivation. Proc Natl Acad Sci USA

| **Field** | **Source-specific context** |
| --- | --- |
| Population / ascertainment | 20 healthy adults. |
| Measurement / assay | Amyloid PET before/after one night of sleep deprivation. |
| Design / comparator | Acute within-person experiment. |
| Reported finding | Regional PET signal changed after deprivation. |
| Reason for inclusion / limit | PET signal change is not proof of newly formed permanent plaques or durable clinical harm. |
| Original source | https://doi.org/10.1073/pnas.1721694115 |

## Reference 34: Holth JK, Fritschi SK, Wang C, Pedersen NP, Cirrito JR, Mahan TE, et al. The sleep–wake cycle regulates brain interstitial fluid tau in mice and CSF tau in humans. Science

| **Field** | **Source-specific context** |
| --- | --- |
| Population / ascertainment | Mouse experiments and a human acute-CSF component; analyze separately. |
| Measurement / assay | Mouse interstitial-fluid tau; human CSF tau under sleep–wake manipulation. |
| Design / comparator | Translational experiments. |
| Reported finding | Tau measures were sensitive to sleep–wake state. |
| Reason for inclusion / limit | Explicit species/compartment distinction; no pooled human therapeutic inference. |
| Original source | https://doi.org/10.1126/science.aav2546 |

## Reference 40: Bubu OM, Pirraglia E, Andrade AG, Sharma RA, Gimenez-Badia S, Umasabor-Bubu OQ, et al. Obstructive sleep apnea and longitudinal Alzheimer’s disease biomarker changes. Sleep

| **Field** | **Source-specific context** |
| --- | --- |
| Population / ascertainment | ADNI clinical cognitive groups; reported OSA exposure. |
| Measurement / assay | Amyloid PET and CSF Aβ42, total tau and phosphorylated tau trajectories. |
| Design / comparator | Observational exposure analysis. |
| Reported finding | OSA associated with selected longitudinal biomarker changes. |
| Reason for inclusion / limit | Measured outcomes are not proof that every participant is biomarker-positive; not randomized PAP efficacy. |
| Original source | https://doi.org/10.1093/sleep/zsz048 |

## Reference 56: Osorio RS, Ducca EL, Wohlleber ME, Tanzi EB, Gumb T, Twumasi A, et al. Orexin-A is associated with increases in cerebrospinal fluid phosphorylated-tau in cognitively normal elderly subjects. Sleep

| **Field** | **Source-specific context** |
| --- | --- |
| Population / ascertainment | Cognitively normal older participants, not an AD-dementia treatment cohort. |
| Measurement / assay | CSF orexin-A and phosphorylated tau. |
| Design / comparator | Human observational biomarker association. |
| Reported finding | Orexin-A associated with CSF phosphorylated tau. |
| Reason for inclusion / limit | Orexin–p-tau association in cognitively normal older adults; CSF orexin does not confirm AD. |
| Original source | https://doi.org/10.5665/sleep.5846 |

## Reference 57: Lozano-Tovar S, Cremascoli R, Nuccetelli M, Sancesario G, Cattaldo S, Prina E, et al. Cerebrospinal-fluid orexin-A levels in different neurocognitive disorders: A comparison study. Neurol Sci

| **Field** | **Source-specific context** |
| --- | --- |
| Population / ascertainment | Different clinically characterized neurocognitive disorders. |
| Measurement / assay | CSF orexin-A. |
| Design / comparator | Cross-diagnostic comparison. |
| Reported finding | Orexin differed by diagnostic context. |
| Reason for inclusion / limit | Orexin is an arousal-related readout, not an interchangeable amyloid or tau marker. |
| Original source | https://doi.org/10.1007/s10072-025-08148-0 |

## Reference 63: Fultz NE, Bonmassar G, Setsompop K, Stickgold RA, Rosen BR, Polimeni JR, et al. Coupled electrophysiological, hemodynamic, and cerebrospinal fluid oscillations in human sleep. Science

| **Field** | **Source-specific context** |
| --- | --- |
| Population / ascertainment | Human sleep physiology participants. |
| Measurement / assay | EEG, hemodynamic fMRI and CSF oscillation signals. |
| Design / comparator | Noninterventional coupled physiological measurement. |
| Reported finding | Oscillations are temporally coupled during sleep. |
| Reason for inclusion / limit | No direct net-solute-clearance or amyloid-treatment endpoint. |
| Original source | https://doi.org/10.1126/science.aax5440 |

## Reference 64: Miao A, Luo T, Hsieh B, Edge CJ, Gridley M, Wong RTC, et al. Brain clearance is reduced during sleep and anesthesia. Nat Neurosci

| **Field** | **Source-specific context** |
| --- | --- |
| Population / ascertainment | Mice under waking, sleep and anesthesia conditions. |
| Measurement / assay | Tracer-based parenchymal clearance approach. |
| Design / comparator | Experimental animal study. |
| Reported finding | Measured clearance was reduced during sleep/anesthesia. |
| Reason for inclusion / limit | Conflicting source retained; compare route, state and measured process, not a universal glymphatic direction. |
| Original source | https://doi.org/10.1038/s41593-024-01638-y |

## Reference 65: Hauglund NL, Andersen M, Tokarska K, Radovanovic T, Kjaerby C, Sørensen FL, et al. Norepinephrine-mediated slow vasomotion drives glymphatic clearance during sleep. Cell

| **Field** | **Source-specific context** |
| --- | --- |
| Population / ascertainment | Mice during sleep-related noradrenergic manipulation/measurement. |
| Measurement / assay | Norepinephrine, slow vasomotion and fluid transport. |
| Design / comparator | Animal mechanistic experiment. |
| Reported finding | Slow vasomotion linked to sleep-related transport. |
| Reason for inclusion / limit | Different tracer/physiology endpoint from Miao and human CSF coupling. |
| Original source | https://doi.org/10.1016/j.cell.2024.11.027 |

## Reference 67: Lyckenvik T, Olsson M, Forsberg M, Wasling P, Zetterberg H, Hedner J, et al. Sleep reduces CSF concentrations of beta-amyloid and tau: A randomized crossover study in healthy adults. Fluids Barriers CNS

| **Field** | **Source-specific context** |
| --- | --- |
| Population / ascertainment | Healthy-adult randomized crossover physiology. |
| Measurement / assay | CSF Aβ and tau concentrations across sleep conditions. |
| Design / comparator | Acute human crossover study. |
| Reported finding | Sleep affected CSF concentrations. |
| Reason for inclusion / limit | Concentration is not a direct rate of long-term solute elimination. |
| Original source | https://doi.org/10.1186/s12987-025-00698-x |

## Reference 66: Eide PK, Vinje V, Pripp AH, Mardal KA, Ringstad G. Sleep deprivation impairs molecular clearance from the human brain. Brain

| **Field** | **Source-specific context** |
| --- | --- |
| Population / ascertainment | Clinical intrathecal MRI sample: 24 participants; 7 sleep-deprived and 17 comparison participants. |
| Measurement / assay | Intrathecal gadobutrol MRI with serial tissue tracer retention. |
| Design / comparator | Human sleep-deprivation comparison. |
| Reported finding | Greater tracer retention after sleep deprivation. |
| Reason for inclusion / limit | Direct human tracer context, but selected clinical population and retention endpoint limit broad extrapolation. |
| Original source | https://doi.org/10.1093/brain/awaa443 |

## Reference 70: Ancoli-Israel S, Palmer BW, Cooke JR, Corey-Bloom J, Fiorentino L, Natarajan L, et al. Cognitive effects of treating obstructive sleep apnea in Alzheimer’s disease: A randomized controlled study. J Am Geriatr Soc

| **Field** | **Source-specific context** |
| --- | --- |
| Population / ascertainment | 52 patients with clinical mild-to-moderate AD and OSA. |
| Measurement / assay | Therapeutic versus sham CPAP; cognitive testing. |
| Design / comparator | Randomized initial 3-week comparison, followed by therapeutic exposure. |
| Reported finding | Primary between-group cognitive advantage not significant; pooled pre–post findings favorable/exploratory. |
| Reason for inclusion / limit | Randomized and within-group conclusions separated in Table 4. |
| Original source | https://doi.org/10.1111/j.1532-5415.2008.01934.x |

## Reference 44: Shieu MM, Zaheed AB, Shannon C, Chervin RD, Conceicao A, Paulson HL, et al. Positive airway pressure and cognitive disorders in adults with obstructive sleep apnea: A systematic review of the literature. Neurology

| **Field** | **Source-specific context** |
| --- | --- |
| Population / ascertainment | 11 studies with differing designs and cognitive populations. |
| Measurement / assay | PAP use/adherence and cognitive disorder outcomes. |
| Design / comparator | Published systematic review; not a new pooled analysis in this manuscript. |
| Reported finding | Promising but heterogeneous evidence; observational and trial results are distinct. |
| Reason for inclusion / limit | Published systematic-review context; broader context, not a vote count establishing efficacy. |
| Original source | https://doi.org/10.1212/WNL.0000000000200383 |

## Reference 41: Lal C, Ayappa I, Ayas N, Beaudin AE, Hoyos C, Kushida CA, et al. The link between obstructive sleep apnea and neurocognitive impairment: an official American Thoracic Society workshop report. Ann Am Thorac Soc

| **Field** | **Source-specific context** |
| --- | --- |
| Population / ascertainment | OSA/cognition literature across adult populations. |
| Measurement / assay | Clinical physiology, treatment and measurement questions. |
| Design / comparator | Official ATS workshop report. |
| Reported finding | Identifies heterogeneity in hypoxemia, arousals, baseline cognition and exposure. |
| Reason for inclusion / limit | Official ATS workshop context; informs clinical modifiers, not a new AD trial. |
| Original source | https://doi.org/10.1513/AnnalsATS.202205-380ST |

## Reference 72: Richards KC, Gooneratne N, Dicicco B, Hanlon A, Moelter S, Onen F, et al. CPAP adherence may slow 1-year cognitive decline in older adults with mild cognitive impairment and apnea. J Am Geriatr Soc

| **Field** | **Source-specific context** |
| --- | --- |
| Population / ascertainment | MCI + OSA; 54 one-year completers, 29 adherent and 25 nonadherent. |
| Measurement / assay | Device use ≥4 versus <4 h/night. Primary cognitive outcome: Hopkins Verbal Learning Test–Revised memory; secondary cognitive outcome: Digit Symbol processing speed. |
| Design / comparator | Nonrandomized adherence comparison. |
| Reported finding | Adjusted secondary processing-speed estimate 1.68 (95% CI 0.73–2.62) favored adherers. This is not the primary memory endpoint or a randomized between-group treatment effect. |
| Reason for inclusion / limit | Direct MCI adherence context; identifies the secondary endpoint and preserves selection/healthy-adherer limitations rather than implying established memory benefit. |
| Original source | https://doi.org/10.1111/jgs.15758 |

## Reference 73: Xu H, Liu Y, Li C, Li X, Shen L, Wang H, et al. Effects of continuous positive airway pressure on neuroimaging biomarkers and cognition in adult obstructive sleep apnea: a randomized controlled trial. Am J Respir Crit Care Med

| **Field** | **Source-specific context** |
| --- | --- |
| Population / ascertainment | 148 cognitively normal adults with moderate-to-severe OSA. |
| Measurement / assay | CPAP + supportive care versus supportive care; MoCA and MRI. |
| Design / comparator | Randomized trial; 6-month primary endpoint. |
| Reported finding | MoCA difference −.04 (95% CI −.72 to .65); MRI outcomes differed. |
| Reason for inclusion / limit | Non-AD counterexample only; shortened narrative and no AD biomarker claim. |
| Original source | https://doi.org/10.1164/rccm.202406-1170OC |

## Reference 75: Herring WJ, Ceesay P, Snyder E, Bliwise D, Budd K, Hutzelmann J, et al. Polysomnographic assessment of suvorexant in patients with probable Alzheimer’s disease dementia and insomnia: A randomized trial. Alzheimers Dement

| **Field** | **Source-specific context** |
| --- | --- |
| Population / ascertainment | 285 patients with probable mild-to-moderate AD dementia and insomnia. |
| Measurement / assay | Suvorexant versus placebo; PSG total sleep time. |
| Design / comparator | 4-week randomized trial. |
| Reported finding | Between-group TST gain 28 min (95% CI 11–45); somnolence 4.2% vs 1.4%; falls in 3/142 (2.1%) vs 0/143. Reported events do not by themselves establish drug causation. |
| Reason for inclusion / limit | Drug-/population-specific symptomatic efficacy, not long-term cognition or class-wide disease modification. |
| Original source | https://doi.org/10.1002/alz.12035 |

## Reference 76: Lucey BP, Liu H, Toedebusch CD, Freund D, Redrick T, Chahin SL, et al. Suvorexant acutely decreases tau phosphorylation and Aβ in the human CNS. Ann Neurol

| **Field** | **Source-specific context** |
| --- | --- |
| Population / ascertainment | 38 cognitively unimpaired middle-aged adults. |
| Measurement / assay | Suvorexant/placebo; serial CSF Aβ and site-specific tau phosphorylation. |
| Design / comparator | Acute randomized pharmacodynamic experiment. |
| Reported finding | Selected CSF Aβ and tau-phosphorylation measures changed. |
| Reason for inclusion / limit | Human target engagement distinguished from the clinical insomnia trial and long-term AD outcomes. |
| Original source | https://doi.org/10.1002/ana.26641 |

## Reference 74: McCurry SM, Gibbons LE, Logsdon RG, Vitiello MV, Teri L. Nighttime insomnia treatment and education for Alzheimer's disease: a randomized, controlled trial. J Am Geriatr Soc

| **Field** | **Source-specific context** |
| --- | --- |
| Population / ascertainment | 36 community AD patient–caregiver dyads. |
| Measurement / assay | Caregiver behavioral education, walking and light; wrist activity-derived sleep. |
| Design / comparator | Randomized program/control; 2-month and 6-month assessments. |
| Reported finding | Nocturnal sleep measures improved in the multicomponent arm. |
| Reason for inclusion / limit | Dementia-adapted care example; no attribution to each component or standard CBT-I equivalence. |
| Original source | https://doi.org/10.1111/j.1532-5415.2005.53252.x |

## Reference 48: Richards KC, Fry LM, Lozano AJ, Ji W, Morrison JD, Britt KC, et al. Treatment of restless legs syndrome improves agitation and sleep in persons with dementia: A randomized trial. J Am Med Dir Assoc

| **Field** | **Source-specific context** |
| --- | --- |
| Population / ascertainment | 147 older adults with clinical AD, agitation and RLS. |
| Measurement / assay | Gabapentin enacarbil versus placebo; agitation and actigraphic sleep. |
| Design / comparator | Syndrome-selected randomized trial. |
| Reported finding | Agitation/sleep outcomes improved; adverse events and nonsignificant falls signal reported. |
| Reason for inclusion / limit | Do not generalize to all insomnia or undifferentiated agitation. |
| Original source | https://doi.org/10.1016/j.jamda.2025.105485 |

## Reference 77: Figueiro MG, Hunter CM, Higgins P, Hornick T, Jones GE, Plitnick B, et al. Tailored lighting intervention for persons with dementia and caregivers living at home. Sleep Health

| **Field** | **Source-specific context** |
| --- | --- |
| Population / ascertainment | Persons with dementia and caregivers living at home. |
| Measurement / assay | Tailored lighting intervention and sleep/care outcomes. |
| Design / comparator | Feasibility/intervention study. |
| Reported finding | Supports feasibility and potential benefit under delivered light conditions. |
| Reason for inclusion / limit | Caregiver context and exposure matter; not an AD disease-modification trial. |
| Original source | https://doi.org/10.1016/j.sleh.2015.09.003 |

## Reference 78: Cremascoli R, Sparasci D, Giusti G, Cattaldo S, Prina E, Roveta F, et al. Effects of circadian phase tailored light therapy on sleep, mood, and cognition in Alzheimer's disease: Preliminary findings in a pivotal study. Front Physiol

| **Field** | **Source-specific context** |
| --- | --- |
| Population / ascertainment | Small AD clinical trial sample. |
| Measurement / assay | Phase-tailored light, circadian timing and clinical outcomes. |
| Design / comparator | Preliminary intervention study. |
| Reported finding | Phase responses and sleep/clinical outcomes require endpoint-specific interpretation. |
| Reason for inclusion / limit | Timing hypothesis and small-sample limits; no blanket evening schedule. |
| Original source | https://doi.org/10.3389/fphys.2021.755322 |

## Reference 79: Leung LYL, Tam HL, Asiamah N, Ho JKM. Effect of melatonin on cognitive function in adults with cognitive impairment: A multi-dimensional meta-analysis of randomized trials. Alzheimers Res Ther

| **Field** | **Source-specific context** |
| --- | --- |
| Population / ascertainment | Heterogeneous cognitively impaired adult trial populations. |
| Measurement / assay | Melatonin; published cognitive-outcome synthesis. |
| Design / comparator | Published meta-analysis. |
| Reported finding | MMSE and ADAS-Cog conclusions differed. |
| Reason for inclusion / limit | Cognitive scales do not override ISWRD-specific guideline recommendations. |
| Original source | https://doi.org/10.1186/s13195-025-01881-w |

## Reference 82: Papalambros NA, Weintraub S, Chen T, Grimaldi D, Santostasi G, Paller KA, et al. Acoustic enhancement of sleep slow oscillations in mild cognitive impairment. Ann Clin Transl Neurol

| **Field** | **Source-specific context** |
| --- | --- |
| Population / ascertainment | Nine participants with aMCI; pathology not uniformly established. |
| Measurement / assay | Closed-loop acoustic stimulation versus sham. |
| Design / comparator | Randomized crossover physiological study. |
| Reported finding | Stimulation-interval SWA increased; whole-night NREM and memory comparisons were not significant. |
| Reason for inclusion / limit | Target engagement and clinical efficacy explicitly separated. |
| Original source | https://doi.org/10.1002/acn3.796 |

## Reference 83: Van den Bulcke L, Davidoff H, Heremans E, Potts Y, Vansteelandt K, De Vos M, et al. Acoustic stimulation to improve slow-wave sleep in Alzheimer’s disease: a multiple night at-home intervention. Am J Geriatr Psychiatry

| **Field** | **Source-specific context** |
| --- | --- |
| Population / ascertainment | 15 patients with AD completing multi-night home protocol. |
| Measurement / assay | Baseline nights, then randomized stimulation/sham nights; SWA/SWS. |
| Design / comparator | Small open-label at-home intervention with within-person night comparisons. |
| Reported finding | Physiological enhancement; durable cognitive benefit not established. |
| Reason for inclusion / limit | Feasibility and physiology, not a large parallel clinical-efficacy trial. |
| Original source | https://doi.org/10.1016/j.jagp.2024.07.002 |

## Reference 84: Zeller CJ, Wunderlin M, Wicki K, Teunissen CE, Nissen C, Züst MA, et al. Multi-night acoustic stimulation is associated with better sleep, amyloid dynamics, and memory in older adults with cognitive impairment. Geroscience

| **Field** | **Source-specific context** |
| --- | --- |
| Population / ascertainment | 18 cognitively healthy older adults and 16 with screening-defined cognitive impairment (MoCA ≤25); not a uniformly biomarker-confirmed AD cohort. |
| Measurement / assay | Multi-night acoustic stimulation, sleep, amyloid-related and memory measures. |
| Design / comparator | One baseline sham night followed by three stimulation nights; response associations and cognitive follow-up, not a parallel randomized efficacy comparison. |
| Reported finding | Response associations with memory do not equal a randomized durable clinical benefit. |
| Reason for inclusion / limit | Selected experimental contrast; preserve comparator and responder-analysis limits. |
| Original source | https://doi.org/10.1007/s11357-024-01195-z |

## Reference 85: Wunderlin M, Züst MA, Hertenstein E, Fehér KD, Schneider CL, Klöppel S, et al. Modulating overnight memory consolidation by acoustic stimulation during slow-wave sleep: a systematic review and meta-analysis. Sleep

| **Field** | **Source-specific context** |
| --- | --- |
| Population / ascertainment | 10 primary studies with 11 experiments and 177 healthy participants; not an AD/MCI treatment meta-analysis. |
| Measurement / assay | Memory outcomes during slow-wave stimulation protocols. |
| Design / comparator | Systematic review/meta-analysis. |
| Reported finding | Overall memory Hedges’ g=0.25, P=.07; selected subgroup estimates were significant. These findings cannot be assigned to an AD population. |
| Reason for inclusion / limit | Preserves the healthy-participant inclusion criterion, the nonsignificant overall result, and the difference between subgroup evidence and AD clinical efficacy. |
| Original source | https://doi.org/10.1093/sleep/zsaa296 |

## Reference 51: Hsieh CF, Tseng PT, Chen TY, Lin PY, Chen YW, Ho BL, et al. The association of changes of sleep architecture related to donepezil: a systematic review and meta-analysis. J Formos Med Assoc

| **Field** | **Source-specific context** |
| --- | --- |
| Population / ascertainment | 12 published donepezil sleep-architecture studies. |
| Measurement / assay | PSG stages, continuity and donepezil exposure. |
| Design / comparator | Published systematic review/meta-analysis. |
| Reported finding | REM percentage increases; some analyses show lower efficiency/TST or stage-2 percentage. |
| Reason for inclusion / limit | Medication-effects synthesis; architecture change is not uniformly better sleep. |
| Original source | https://doi.org/10.1016/j.jfma.2021.10.013 |

## Reference 50: Carvalho DZ, St Louis EK, Knopman DS, Boeve BF, Lowe VJ, Roberts RO, et al. Association of excessive daytime sleepiness with longitudinal β-amyloid accumulation in elderly persons without dementia. JAMA Neurol

| **Field** | **Source-specific context** |
| --- | --- |
| Population / ascertainment | 283 older adults without dementia. |
| Measurement / assay | Baseline sleepiness; repeated amyloid PET. |
| Design / comparator | Longitudinal observational study. |
| Reported finding | Sleepiness associated with regional subsequent amyloid accumulation. |
| Reason for inclusion / limit | Longitudinal amyloid-PET context; not a trial of treating sleepiness. |
| Original source | https://doi.org/10.1001/jamaneurol.2018.0049 |

## Reference 35: Rongve A, Boeve BF, Aarsland D. Frequency and correlates of caregiver-reported sleep disturbances in a sample of persons with early dementia. J Am Geriatr Soc

| **Field** | **Source-specific context** |
| --- | --- |
| Population / ascertainment | 151 early-dementia participants of mixed etiologies, plus 420 controls. |
| Measurement / assay | Caregiver Mayo Sleep Questionnaire and NPI insomnia report. |
| Design / comparator | Cross-sectional symptom prevalence. |
| Reported finding | 71% any sleep disturbance; insomnia 29.9%, EDS 22.6% in dementia sample. |
| Reason for inclusion / limit | Burden context; reported/probable symptoms are not PSG-confirmed AD-specific prevalence. |
| Original source | https://doi.org/10.1111/j.1532-5415.2010.02733.x |

## Reference 36: Gaeta AM, Benítez ID, Jorge C, Torres G, Dakterzada F, Minguez O, et al. Prevalence of obstructive sleep apnea in Alzheimer's disease patients. J Neurol

| **Field** | **Source-specific context** |
| --- | --- |
| Population / ascertainment | 128 selected clinical AD patients. |
| Measurement / assay | PSG AHI and Epworth sleepiness scale. |
| Design / comparator | Cross-sectional clinic prevalence. |
| Reported finding | 90.6% had AHI>5/h; median Epworth score 5. |
| Reason for inclusion / limit | Clinic/threshold context; little subjective sleepiness does not exclude OSA. |
| Original source | https://doi.org/10.1007/s00415-019-09668-4 |
